# Hydrogen Production by the Ruthenium(II) Complex Bearing a Bulky PNP Ligand: A Catalyst for the Decomposition of Formic Acid and/or Ammonium Formate

**DOI:** 10.1021/acsomega.4c09025

**Published:** 2024-12-11

**Authors:** André L. Bogado, Leon Kambiz Paschai Darian, David Bürgy, Lucas da Silva dos Santos, Leonardo Tsuyoshi Ueno

**Affiliations:** † Instituto de Ciências Exatas e Naturais do Pontal, Universidade Federal de Uberlândia, Ituiutaba, Minas Gerais CEP 38304-402, Brazil; ‡ Anorganisch-Chemisches Institut, Universität Heidelberg, Im Neuenheimer Feld 270, Heidelberg 69120, Germany; § Instituto de Química, Universidade Federal de Uberlândia, Uberlândia, Minas Gerais CEP 38400-902, Brazil; ∥ Departamento de Química, Instituto Tecnológico de Aeronáutica, São José dos Campos, São Paulo CEP 12228-900, Brazil

## Abstract

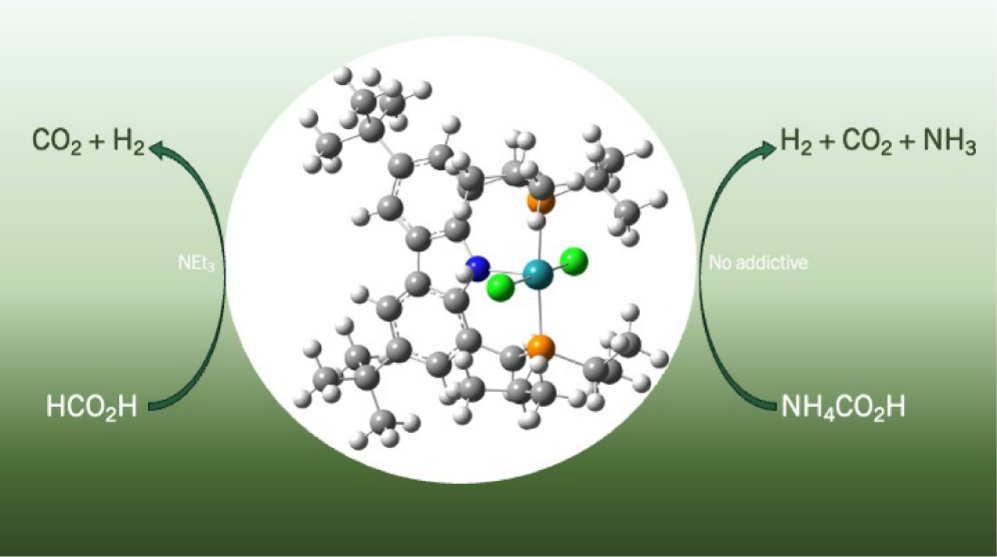

The five-coordinate complex [RuCl_2_(PNP)] (**1**) was synthesized from the binuclear [RuCl_2_(*p*-cym)]_2_ with a PNP-type ligand (PNP = 3,6-di-*tert*-butyl-1,8-bis(di*iso*propylphosphino)methyl)-9*H*-carbazole – (Cbzdiphos*^i^*^Pr^)H) in a toluene solution, within 20 h at 110 °C, producing a green solid, which was precipitated with a 1/1 mixture of *n*- pentane/HMDSO. The complex was characterized by NMR—^1^H, ^13^C, and ^31^P{^1^H}, mass spectroscopy—LIFDI, FTIR, UV/vis spectroscopy, and cyclic voltammetry, as well as a description of the optimized structure by DFT calculation. The reactivity of **1** was investigated in the presence of potassium triethylborohydride (KBEt_3_H, in THF solution of 1.0 mol L^–1^) and ammonium formate (NH_4_HCO_2_), producing an *in situ* hydride complex and a formate intermediate species coordinated to the ruthenium center. The complex **1**, loaded with 0.08%, catalyzed the decomposition of ammonium formate (AF) into H_2_, CO_2_, and NH_3_ in THF solutions at 80 °C, with 94% of H_2_ and TOF = 206 h^–1^ (molar ratio [Ru]/AF = 1/1204). The catalytic activity increased remarkably for the decomposition of formic acid (FA) as a substrate to produce H_2_ and CO_2_. In the HMDSO solution at 80 °C, a conversion of 100% was obtained in relation to H_2_ and TOF = 3010 h^–1^ (molar ration [Ru]/FA/NEt_3_ = 1/1204/843). In an equimolar mixture of AF/FA in HMDSO solution at 80 °C, without additives, the complex **1** catalyzed the decomposition of both with 100% of H_2_ and TOF = 987 h^–1^ (molar ratio [Ru]/AF/FA= 1/602/602). Under the later conditions, as well as upon AF decomposition, carbamic acid [HO(C=O)NH_2_] was obtained as a coproduct of a secondary reaction between NH_3_ and CO_2_ (yield = 50% in relation to the amount of AF). A kinetic study for decomposing FA, in the range of 60–100 °C, provided Δ*S*‡ = −9.7 e.u, Δ*G*‡ = 13.35 kJ mol^–1^, and *E*_a_ = 64 kJ mol^–1^, suggesting that the mechanism is more associative than for the known complexes.

## Introduction

The process of decomposition of formic acid (FA) with a heterogeneous catalyst has been known for over 100 years,^1^ but only in the 1960s, scientists investigated this reaction using homogeneous catalysts, which are more selective and prevent the formation of carbon monoxide (CO). In this context, FA can be decomposed following two pathways: a) a more spontaneous reaction producing CO_2_ + H_2_ (Δ*G*° = −38.3 kJ mol^–1^) and b) a less spontaneous reaction producing CO + H_2_O (Δ*G*° = −9.66 kJ mol^–1^).^2^

The second reaction is highly undesired, when a coordination compound is involved in the system as catalyst or precatalyst, since small amounts of CO can deactivate the catalytic species.^2^ Only in 2008, Beller^3^ and Laurenczy^4^ described independently the use of ruthenium complexes as catalysts for the dehydrogenation of FA without CO release. Two years later, Dupont and coworkers^5^ showed the use of [{RuCl_2_(*p*-cymene)}_2_] dissolved in the ionic liquid, without additional bases, to promote the decomposition of FA without CO with remarkable catalytic activity observed during recycles. Since then, many metal-based catalysts have been tested for this purpose: Fe,^6,7^ Co,^8^ Ru,^9−11^ Ir,^12^ Pd,^13^ Mo,^14^ Rh,^15^ and Ni.^16^

The decomposition products of FA (H_2_ + CO_2_) can be used directly in chemical processes or captured and recycled to regenerate it.^17,18^ The molecular hydrogen (H_2_) produced can also be exploited in a fuel cell,^2^ which is considered one of the most important sources of energy, besides being free of pollutant emissions. Alternatively, the catalytic decomposition of FA to generate syngas (a mixture of H_2_ and CO) is also a valuable strategy for energy conversion, since it can be used directly in internal combustion engines or converted to liquid fuels.^19^

Considering the catalytic decomposition of FA with ruthenium-based catalysts, a variety of ligand cases have been used successfully.^20−23^ These include sulfonated phosphins,^24^ α-diimines,^9^*N*-donor chelating ligands,^25^*N*-heterocyclic carbenes,^16^ and [PNP]-pincer ligands.^11^ Related to the last example, by combining a *N*-heterocycle with two phosphine donors, two classes of *N*-heterocyclic PNP ligands are known: (i) an uncharged protonated or tertiary PNP ligand and (ii) an anionic PNP pincer via deprotonation of the neutral ligand.^26,27^ The effect of PNP donor ligands coordinated to ruthenium with a NH and *N*-methylated moiety on the catalytic dehydrogenation of formic acid is documented in the currently literature.^11^ In all cases, the catalyst containing the N-Me portion was superior to the complex containing N–H.

Another interesting and efficient source of molecular hydrogen is ammonium formate (AF), since formates store H_2_ in chemical bonds using the concept of H_2_ carriers.^28^ Alkali metal formates can decompose to H_2_, carbonates (M_2_CO_3_), and bicarbonates (MHCO_3_) in the presence of water or methanol as solvent,^29^ and in the absence of water and an appropriate catalyst, they can produce H_2_, CO_2_, and NH_3_.^30^

Some examples of catalytic systems for the decomposition of AF are described in the literature using heterogeneous catalysts: Pd/C,^28−31^ Au_3_Pd_1_,^32^ Pd nanoparticles (Pd/CS-GO),^33^ and Au/TiO_2_.^34^ The results are relevant in the context of efficient generation of H_2_ from hydrogen-carrying organic molecules.

To the best of our knowledge, in the last 24 years, only one example using a homogeneous Pd catalyst has been reported for this purpose.^29^ Many homogeneous catalysts could be adapted to investigate the dehydrogenation of ammonium formate. For example, Mellmann^35^ and Xu^36^ discuss homogeneous catalysts for the dehydrogenation of formic acid and discuss conditions and catalysts that could be applicable also to derivative salts such as ammonium formate. Here, we present a system that employs a homogeneous catalyst for this reaction, which, to our knowledge, is the first example that uses a ruthenium coordination complex.

This work describes the synthesis and characterization of the complex [RuCl_2_(PNP)](**1**), the chemical reactivity toward KBEt_3_H and NH_4_HCO_2_, and the application of **1** as a catalyst for the homogeneous decomposition of a mixture of FA, AF, and FA/AF.

## Experimental Section

All manipulations were carried out under an inert atmosphere of dry argon (Argon 5.0, purchased from Messer Group GmbH and dried over Granusic granulated phosphorus pentoxide) using standard Schlenk techniques or by working in a glovebox. Solvents were dried according to literature procedures.^37^ The ligands (Cbzdiphos*^i^*^Pr^)H^38^ and [RuCl(H)(PPh_3_)_3_]^39,40^ were synthesized according to the literature. All other chemicals were purchased and used as received without further purification. NMR spectra were recorded on Bruker Avance III (600 MHz) and Bruker Avance II (400 MHz) instruments. Chemical shifts (δ) are given in parts per million (ppm) and are referenced to residual proton or the carbon resonance solvent signals.^41^ H_3_PO_4_ (^31^P) was used as an external standard for the ^31^P{^1^H} NMR data. The following abbreviations were used: s (singlet), d (doublet), t (triplet), m (multiplet), sept (septet), and br (broad signal). Mass spectra were acquired on a JEOL JMS-700 magnetic sector (LIFDI) spectrometer at the mass spectrometry facility of the Institute of Organic Chemistry, the University of Heidelberg. UV/vis spectroscopy was performed on a Varian instrument, model Cary 5000, in the range of 1500–220 nm, using a quartz cell with 1 cm of optical length, which was prepared for measurements under an argon atmosphere. FTIR-ATR spectroscopy was recorded in an Agilent Cary 630 instrument in the range of 400–4000 cm^–1^ by using solid-state samples. TGA was performed in a Mettler Toledo TGA2 instrument under a constant flow of a N_2_ atmosphere (50 mL/min), using a heating rate of 10 K/min from 30 to 600 °C. Cyclic voltammetry was carried out in a Potentiostat PAL Sens instrument, model EMStat3+Blue, with Bluetooth connection, inside the glovebox. The electrochemistry cell was built with a vase, containing a THF solution of [RuCl_2_(PNP)] (5 mmol L^–1^), HTBA as a support electrolyte (*n*-Bu_4_NPF_6_, 0.1 mol L^–1^), and three electrodes: a glassy carbon disc (WE), a Pt wire (CE), and a Ag wire (RE). The ground-state structure of [RuCl_2_(PNP)] was optimized by the density functional theory (DFT), using five different functionals, i.e., B3LYP, B3PW91, CAM-B3LYP, LC-wPBE, and PBE0. The basis sets used to build the molecular orbitals were LANL2DZ (Los Alamos National Laboratory 2 double-ζ) for ruthenium, 6-31G for carbon and hydrogen, and 6-31G(d,p) for the remaining atoms. The Hessian matrix was calculated for the optimized structures to verify the nature of the stationary state. All calculations were carried out using Gaussian09.^42^

### Synthesis of [RuCl_2_(PNP)] (**1**)

Inside a glovebox, binuclear [RuCl_2_(*p*-cym)]_2_ (150 mg; 0.245 mmol) was added in a Schlenk tube with toluene (5 mL). The PNP-type ligand 3,6-di-*tert*-butyl-1,8-bis(di*iso*propylphosphino)methyl)-9*H*-carbazole – (Cbzdiphos*^i^*^Pr^)H (277 mg; 0.514 mmol) was added inside the Schlenk tube with an additional amount of toluene (5 mL). The Schlenk tube was closed, and outside the glovebox, the mixture was heated to 110 °C and stirred for 20 h. Afterward, the color changed from orange to green, and the temperature was cooled to room temperature. The solvent was pumped off under a vacuum until dry, and the crude green oil was dried under a vacuum for at least 1 h. The Schlenk tube was opened inside the glovebox, and the residue was washed with a mixture of *n*-pentane/hexamethyldisiloxane (HMDSO, 1/1 v/v), producing a green solid that was filtered into another Schlenk tube using a funnel. The remaining solution was pumped off, outside the glovebox, and dried under a vacuum for another 1 h. This procedure was repeated 3-fold or until no solid was observed after washing with the solvent mixture. Yield 80% (140 mg; 0.196 mmol). ^1^H NMR (600.1 MHz, C_6_D_6_, rt) δ 7.89 (s, 2H, H_Carb_), 7.35 (s br, 1H, H_N_), 7.23 (s, 2H, H_Carb_), 3.39 (d, *J*_HH_ = 13.3 Hz, 2H, C*H*_2_), 3.18 (dt, *J*_HH_ = 13.4 Hz, *J*_PH_ = 3.3 Hz 2H, CH_2_), 2.51–2.47 (m, 2H, C*H*(CH_3_)_2_), 2.13–2.08 (m, 2H, C*H*(CH_3_)_2_), 1.52 (vq, *J* = 6.6 Hz, 6H, CH(C*H*_3_)_2_), 1.45 (vq, *J* = 6.6 Hz, 6H, CH(C*H*_3_)_2_), 1.37 (s, 18H, C(C*H*_3_)_3_), 1.13–1.07 (m, 12H, CH(C*H*_3_)_2_). ^13^C NMR (150.9 MHz, C_6_D_6_, rt) δ (ppm): 148.8 (s, 2C, C_Carb_), 144.5 (t, *J*_PH_ = 3.9 Hz, 2C, C_Carb_), 133.2 (s, 2C, C_Carb_), 126.0 (t, *J*_PH_ = 2.5 Hz, 2C, C_CarbH_), 125.0 (s, 2C, C_Carb_), 115.2 (s, 2C, C_CarbH_), 34.9 (s, 2C, *C*(CH_3_)_3_), 31.8 (s, 6C, C(*C*H_3_)_3_), 26.0 (t, *J*_PH_ = 5.9 Hz, 2C, *C*H_2_), 23.5 (t, *J*_PH_ = 2.3 Hz, 2C, CH(*C*H_3_)_2_), 22.5 (t, *J*_PH_ = 11.0 Hz, 2C, *C*H(CH_3_)_2_), 21.5 (s, 2C, CH(*C*H_3_)_2_), 20.6 (t, *J*_PH_ = 10.0 Hz, 2C, *C*H(CH_3_)_2_), 19.5 (t, *J*_PH_ = 2.1 Hz, 2C, CH(*C*H_3_)_2_), 16.4 (t, *J*_PH_ = 2.2 Hz, 2C, CH(*C*H_3_)_2_). ^31^P{^1^H} NMR (242.9 MHz, C_6_D_6_, rt): δ (ppm): 66.0. ^31^P{^1^H} NMR (242.9 MHz, C_6_D_6_, rt) δ (ppm): 66.0. MS (LIFDI): *m*/*z* (%) calcd. for C_34_H_55_Cl_2_NP_2_Ru^+^ 710.7228, found 710.2271 (100) ([M]^+^). FTIR-ATR (cm^–1^): ν_P–C_ = 879; ν_C=C_ = 1460; ν_C–N_ = 1607; and ν_C-Hsp3_ = 2952. UV/vis (THF solution, 0.5 × 10^–3^–3.1 × 10^–5^ mol L^–1^): λ_max_ (nm), ε_max_ [mol^–1^ L cm^–1^]; 418 (1335); 800 (1262). CV: *E*_pa_ = −0.25; *E*_pc_ = −0.44 V (Δ*E*_p_ = 0.19 V); *I*_pa_/*I*_pc_ = 1.01. Δ*E*_p_ and *I*_pa_/*I*_pc_ values of the [Fc]/Fc]^+^ couple under these conditions are 0.86 V and 0.83.

### Catalysis

#### Decomposition of Ammonium Formate (AF) or Formic Acid (FA)

A standard experiment was carried out in a 50 mL three-way Schlenk tube, which allowed degassing the system with an argon atmosphere, adding or withdrawing chemicals, and releasing gases from the decomposition of AF or FA. Inside the glovebox, the Schlenk tube was loaded with AF (631 mg; 10 mmol), THF (10 mL), and a sample of [RuCl_2_(PNP)] (5.9 mg; 8.3 μmol; 0.08%) previously prepared in THF (1 mL). In the case of FA, the system was loaded with HMDSO (10 mL), FA (0.360 mL; 10 mmol), NEt_3_ (1.0 mL; 7.0 mmol), and the complex [RuCl_2_(PNP)] in the same amount as described in the decomposition of AF. Reactions were magnetically stirred in an oil bath at 80 °C, and the released gases were washed in two traps in the case of AF, one containing concentrated H_2_SO_4_ (100 mL) and other with NaOH (100 mL, 8 mol L^–1^). In the case of FA, the released gases were washed in a trap containing NaOH (100 mL, 8 mol L^–1^). The residual gas, hydrogen, was collected and measured in a 500 mL U-shaped graduated water column at atmospheric pressure. At the end of each run, the graduated water column was depressurized without allowing air to enter.

## Results and Discussion

### Synthesis, General Characterization, and Reactivity

**Scheme 1:**
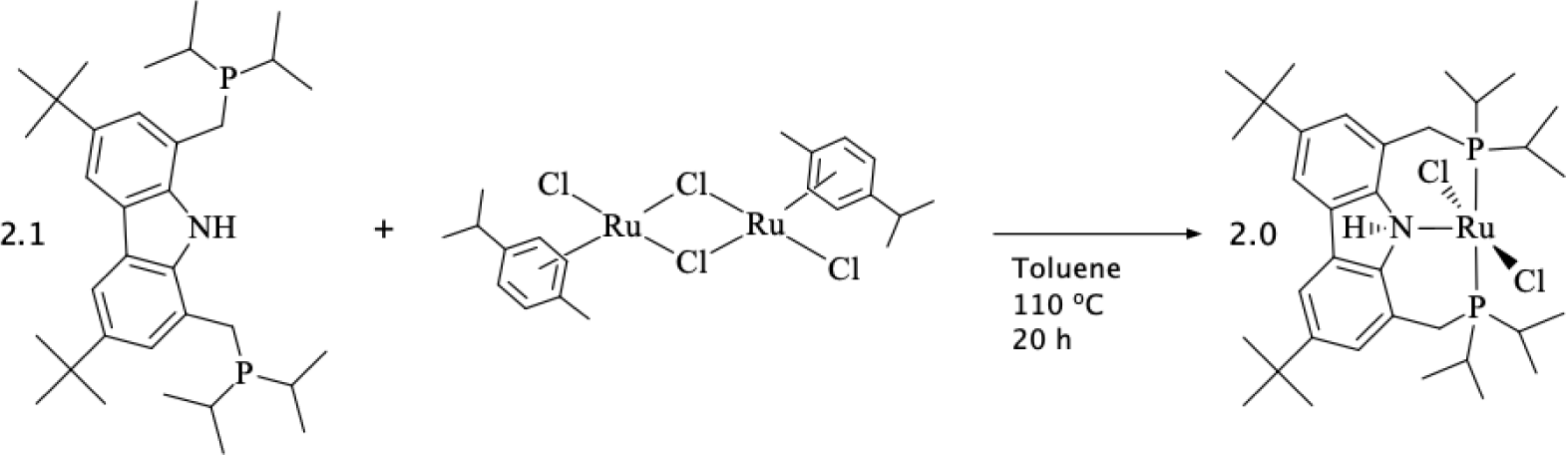
Synthetic Route and General Structure of [RuCl_2_(PNP)] (**1**)

The five-coordinate complex [RuCl_2_(PNP)] was synthesized from the well-known binuclear ruthenium precursor [RuCl_2_(*p*-cym)]_2_ in the presence of 2.1 eq. of the PNP ligand in toluene solution at 110 °C and 20 h {where PNP = 3,6-di-*tert*-butyl-1,8-bis(di*iso*propylphosphino)methyl)-9*H*-carbazole – (Cbzdiphos*^i^*^Pr^)H} (see Scheme 1). The ^1^H and ^13^C NMR (Figures S1 and S2) agree with the structure described in Scheme 1, which is supported by 2D NMR experiments, HSQC (heteronuclear single quantum coherence), and HMBC (heteronuclear multiple bond correlation) (see Figures S3 and S4, respectively). The ^31^P{^1^H} NMR data showed only a singlet signal at 66 ppm, consistent with similar complexes described in literature,^43^ when P atoms are coordinated *trans* to each other in a PNP moiety (Figure S5). FTIR-ATR spectroscopy provided the main stretching bands for PNP coordination in the metal center, such as ν_P–C_ = 879 cm^–1^; ν_C=C_ = 1460 cm^–1^; ν_C–N_ = 1607 cm^–1^; and ν_C-Hsp3_ = 2952 cm^–1^ (Figure S6). Mass spectroscopy (LIFDI) calculated for C_34_H_55_Cl_2_NP_2_Ru^+^ 710.7228 *m*/*z* was found 710.2271 *m*/*z* (100) ([M]^+^) (Figure S7), validating the molecular formula and structure attributed in Scheme 1.

Cyclic voltammetry (CV), which was swept anodically from −1.0 to 0.0 V, revealed an anodic peak potential (*E*_pa_) at −0.25 V and a cathodic peak potential (*E*_pc_) at −0.44 V, due to the Ru^3+^/Ru^2+^ redox pair. It corroborates the strong electron donating effect of the PNP ligand toward the Ru(II) center. The values observed for the potential variation (Δ*E*_p_) and *I*_pa_/*I*_pc_ are lower than the values observed for the [Fc]/Fc]^+^ couple under the same conditions {where Fc = ferrocene}. A diffusion-controlled reversible process was confirmed by a linear relationship between current (*I*) versus the square root of the potential sweep rate (see Figure S8).

The UV/vis spectra (Figure S9) scanned from 1500 until 220 nm of [RuCl_2_(PNP)] in THF solution (0.5 × 10^–3^–3.1 × 10^–5^ mol L^–1^) showed three absorption bands in λ_max_ = 418, 491, and 800 nm and molar coefficient (ε_max_) = 1335, 488, and 1262 mol^–1^ L cm^–1^, respectively.

In the presence of KBEt_3_H (10 equiv), complex **1** produces a monohydride complex, which showed a singlet signal with the chemical shift at 45 ppm in the ^31^P{^1^H} NMR spectra. The ^1^H NMR data presented a triplet centered at −9.25 ppm, due to the heteronuclear coupling between the hydride and the P atoms (*J*_HP_ = 16.4 Hz). Then, the NMR tube was heated at 70 °C for 16 h, and the color changed from yellowish orange to yellow. The ^31^P{^1^H} and ^1^H NMR data also changed to a singlet signal at 85 ppm and a multiplet at −9.25 ppm, respectively, suggesting the *in situ* formation of a ruthenium dihydride complex containing a PNP ligand (see Figure S10). A boron adduct at 4.4 ppm was also observed in the ^31^P{^1^H} spectra. The reactivity of **1** in the presence of ammonium formate (AF) produces an *in situ* species observed at 69.8 ppm in the ^31^P{^1^H}, which unshielded the P atoms of the PNP ligand. This result agrees with a replacement of the chlorine ligand by a stronger σ-donor formate ion (^−^OCOH) (Figure S11).

**Figure 1:**
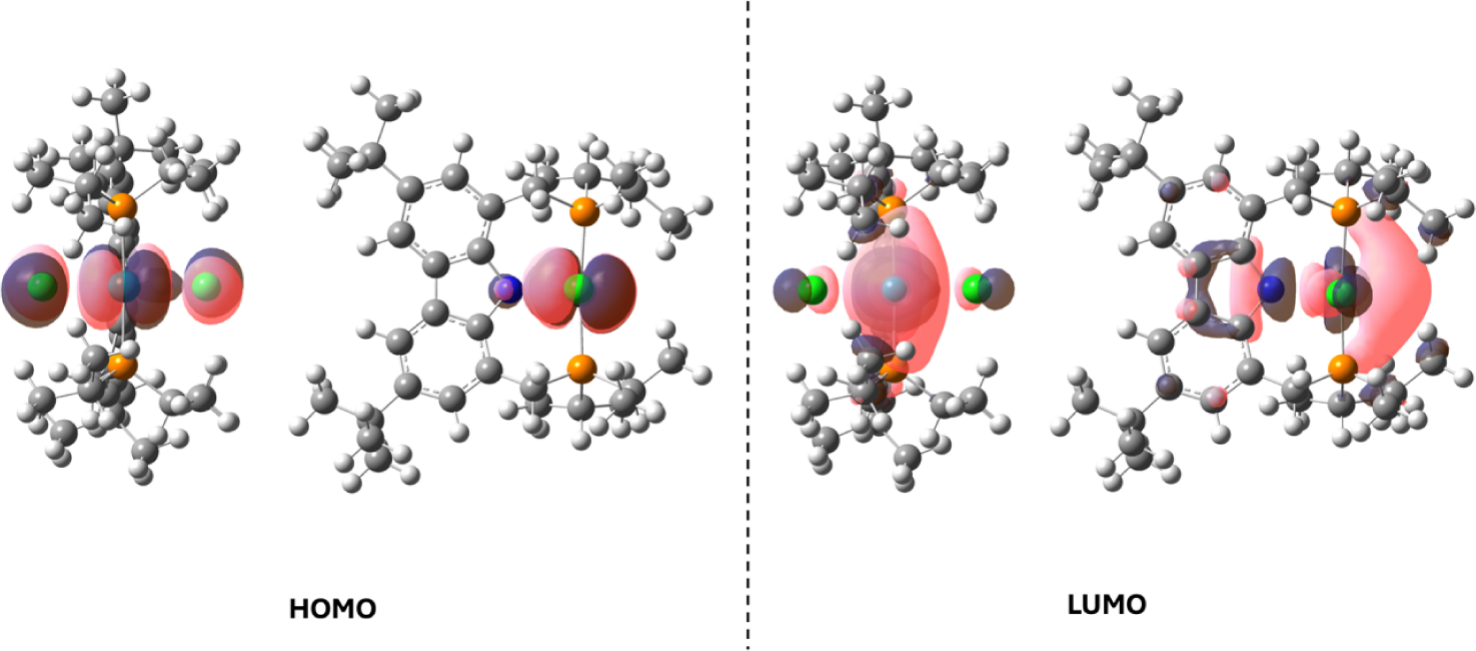
HOMO and LUMO orbitals of the optimized structure of **1**, using the B3PW91 functional in THF. LANL2DZ basis for ruthenium, 6-31G for carbon and hydrogen, and 6-31G(d,p) for the remaining atoms.

Five different functionals were used to optimize the structure of **1** by DFT calculations (see Table S1 and Figure S12). In general, the HOMO and LUMO orbitals obtained from the different functionals were quite similar. The functional base B3PW91 was chosen to describe the optimized structure because it represented intermediate values among the functionals used (Figure 1). The HOMO orbital is in the Cl–Ru–Cl moiety, while the LUMO orbital is positioned toward the carbazole ring, the halogen groups, and the sixth vacant site position. This latter result agrees with the remarkable catalytic activity of **1** in the decomposition of AF and FA, suggesting that an open site position is available for substrates in the core sphere of the complex. It is important to mention that the bulky PNP ligand forces a nearly linear angle to the Cl–Ru–Cl bonds (178°, see Table S1), which could allow a coordination of small species in the sixth position of the open site in an octahedral environment. A nucleophilic attack of the formate anion at the sixth position, from the deprotonation of FA or directly from the AF salt, could initiate the decomposition reaction, producing H_2_ and CO_2_ in the case of FA or H_2_, CO_2_, and NH_3_ in the case of AF.

### Catalysis

**Scheme 2:**
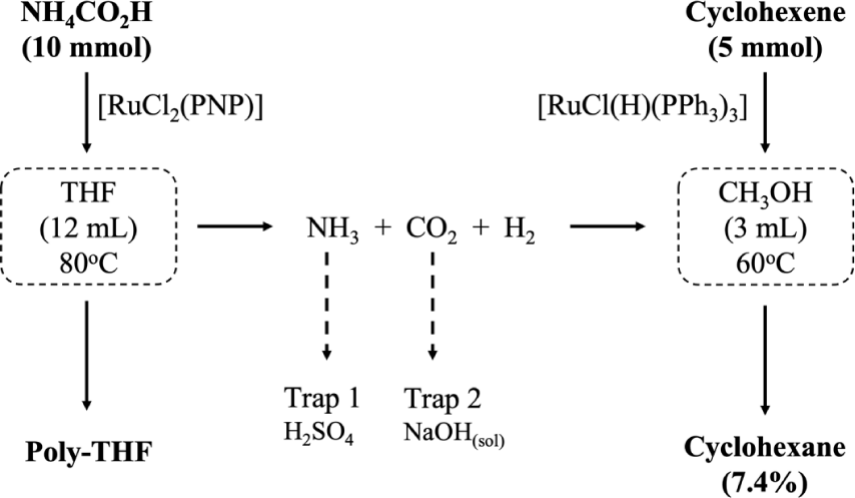
Production of H_2_ from AF and Its Use in the Cyclohexene Hydrogenation Reaction

The complex [RuCl_2_(PNP)] (**1**) catalyzed the decomposition of formic acid (FA) and ammonium formate (AF), separately or in a mixture of both. In a typical experiment for decomposing FA, **1** was loaded with 0.08%, and the released gases passed through a solution of NaOH (100 mL, 8 mol L^–1^) to absorb CO_2_ (Figure S13). In the case of AF decomposition, the gases also passed through a commercial H_2_SO_4_ solution trap (100 mL, 95–98%) to capture NH_3_. The remaining gas was identified as H_2_ by volumetric analysis and in the presence of a hydrogen acceptor (cyclohexene), which was hydrogenated to cyclohexane in the presence of the known complex [RuCl(H)(PPh_3_)_3_] as a catalyst in methanol solution (see Figure S14). Scheme 2 illustrates the production of H_2_ from AF and its use in the cyclohexene hydrogenation reaction.

The decomposition reaction of AF (10 mmol) produced 94% H_2_ (230 mL) in THF solution within 330 min at 80 °C. It represents a turnover number (TON) of 1132 and a turnover frequency (TOF) of 206 h^–1^ in the pseudo-first order reaction with *k*_obs_ = 3.4 × 10^–2^ min^.–1^. H_2_ was used in a parallel reaction, loaded with the complex [RuCl(H)(PPh_3_)_3_] (10 μmol), cyclohexene (5 mmol), and methanol (3 mL) (Figure S14). The H_2_ atmosphere was maintained for 20 h at 60 °C, and the hydrogenation reaction was monitored by ^1^H NMR spectroscopy (Figure S15). Therefore, cyclohexane was obtained in 7.4% yield, and no hydroformylation product was observed. It suggests, at least under these conditions, that the decomposition of AF does not produce carbon monoxide. However, a side reaction was observed as a polymerization of THF, producing a white rubber identified as poly-THF with 15% yield. This protocol was repeated for FA decomposition, and a similar amount of cyclohexane without coproducts was observed.

**Figure 2:**
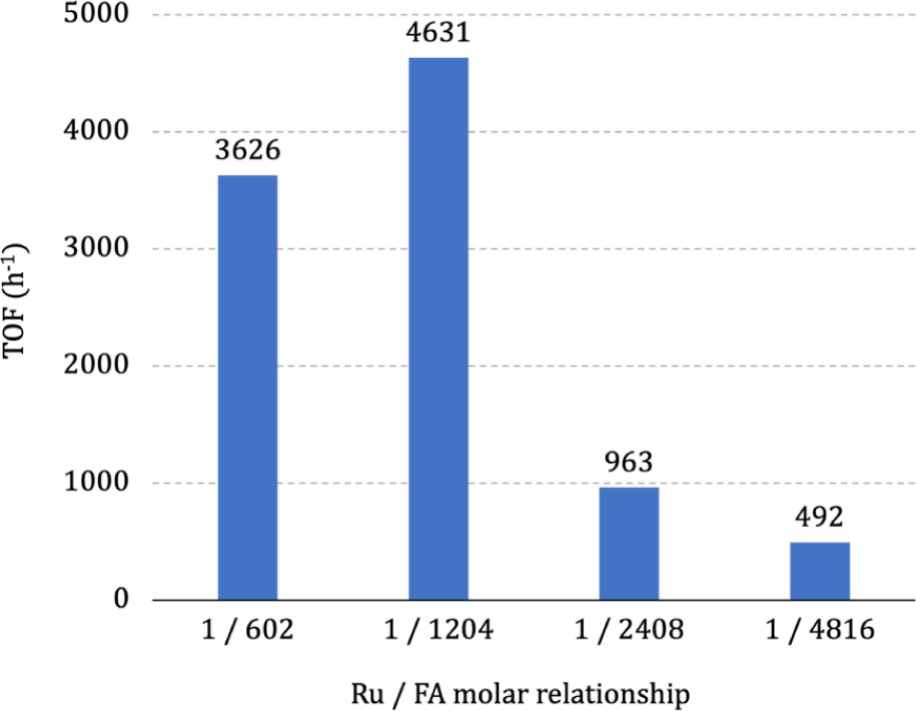
Variation of catalyst loading at 90 °C for FA decomposition in the presence of NEt_3_ (7 mmol) in HMDSO solution (10 mL) containing THF (1 mL).

Varying the catalyst loading from 0.02 to 0.16% at 90 °C showed the best molar ratio for Ru/FA = 1/1204 or 0.08% of the ruthenium complex (Figure 2), which produced H_2_ at the rate of 4631 h^–1^. The amount of NEt_3_ was kept constant at 7 mmol in each catalytic run.

The catalytic activity of **1** in the decomposition of FA increases, reaching a maximum at the molar ratio 1/1204, and then decreases rapidly, due to the large amount of substrate (Figure 2). The best molar ratio for Ru/FA = 1/1204 or 0.08% of the ruthenium complex, producing 100% of H_2_ at a rate of 4631 h^–1^. With a high amount of FA, the complex becomes insoluble under the reaction conditions, reducing its catalytic activity. Therefore, as observed in Figure 2, only 10% of H_2_ was obtained at a 1/4816 catalyst/substrate molar ratio.

Table 1 summarizes the results for the decomposition of FA and AF using [RuCl_2_(PNP)] as a catalyst.

**Table 1:** Decomposition of FA and/or AF Using [RuCl_2_(PNP)] as a Catalyst^a^

Entry	Substrate^a^	Solvent	Base	Temp. (°C)	Time (h)	Volume (mL)	YH_2_ (%)	TON	TOF (h^–1^)
1	AF^b^	THF	---	80	5.0	300	---	---	---
2	AF^b^	THF	---	80	5.5	230	94	1132	206
3	AF^b^	CH_3_OH	---	80	1.0	0	0	0	0
4	AF^b^	HMDSO*	---	80	5.3	160	66	786	149
5	FA^c^	---	NEt_3_	60	3.0	20	4	48	16
6	FA^c^	HMDSO*	NEt_3_	80	0.4	245	100	1204	3010
7	FA^c^	THF	NEt_3_	80	0.8	245	100	1204	1505
8	FA/AF^d^	---	---	60	1.0	0	0	0	0
9	FA/AF^e^	HMDSO*	---	80	1.2	245	100	1204	987

a AF = ammonium formate. FA = formic acid. * 10 mL of HMDSO containing 1 mL of THF.

b Molar ratio: Ru/AF = 1/1204.

c Molar ratio: Ru/FA/base = 1/1204/843.

d Molar ratio: Ru/FA/AF = 1/1204/120.

e Molar ratio: Ru/FA/AF = 1/602/602.

**Figure 3:**
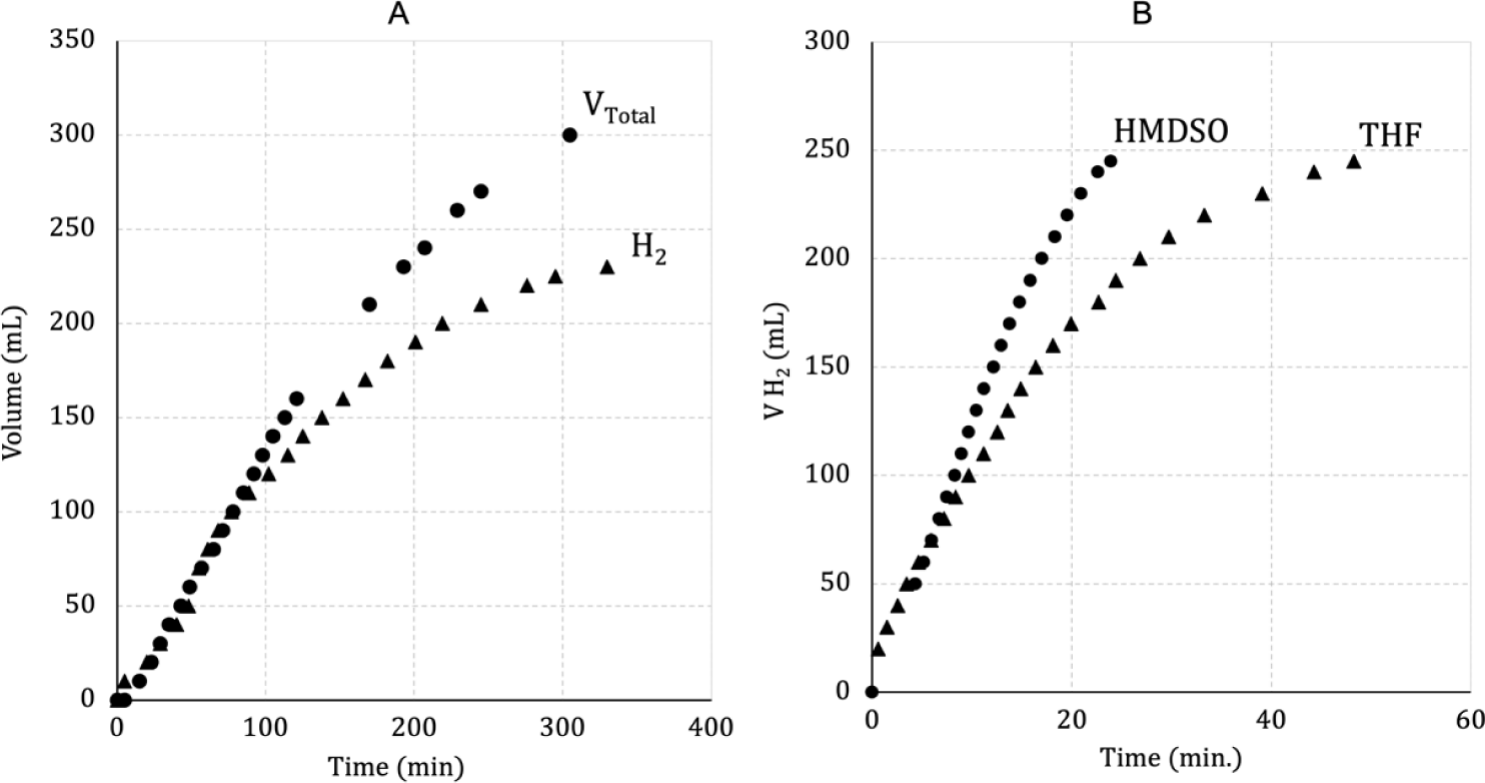
(A) Performance of the complex [RuCl_2_(PNP)] in the decomposition of AF with and without CO_2_ and NH_3_ traps. (B) Effect of the solvent on the catalytic performance of [RuCl_2_(PNP)] in the decomposition FA.

The total amount of gases from the decomposition of AF in THF without traps (entry 1, Table 1) was slightly higher when compared to the reaction with a NaOH and H_2_SO_4_ trap (entry 2, Table 1), suggesting that CO_2_ and NH_3_ might be dissolved in the graduated water column, which was used to measure the amount of gases (Figures 3A and S14). It is important to mention that the maximum amount of H_2_ that could be generated in this protocol is 245 mL at room temperature (25 °C) and 1 atm. To avoid the THF polymerization reaction, methanol was used as a solvent, but in this case, the production of gases was not observed (entry 3). As a consequence of this, hexamethyldisiloxane (HMDSO) was chosen as a solvent, and 66% of H_2_ was obtained (entry 4) without polymers. When entries 2 and 4 are compared, it is evident that THF is the best solvent for AF decomposition, even though a secondary THF polymerization reaction is present.

In this study, decomposition of FA without a solvent (entry 5) produced a small amount of H_2_, due to the low solubility of **1** in FA, and did not work without NEt_3_. To increase the catalytic activity of **1**, HMDSO was added as a solvent (entry 6), producing 100% H_2_ in 24 min at a rate of 3010 h^–1^.

The TOF decreases by half in the THF solution (entry 7), due to the THF polymerization reaction (Figure 3B). The attempt to promote the decomposition of FA/AF without a solvent did not work due to the low solubility of the complex in the reaction medium (entry 8). However, in the presence of HMDSO as a solvent, an equimolar mixture of FA/AF was decomposed, and 100% H_2_ was obtained under mild conditions (entry 9).

In this last reaction, when the system was cooled at room temperature, a white solid was obtained in the reaction flask, which was filtered off and washed with HMDSO (3 × 5 mL), and dried in an air atmosphere. The physicochemical characterization of this white compound suggests the formation of carbamic acid, which can be obtained from the parallel reaction between CO_2_ + NH_3_ (see Figures S16 – S19).

The temperature dependence on the performance of **1** in FA decomposition showed an increase in the rate with increasing temperature in the range of 60–100 °C. Table 2 summarizes the kinetic results for 50% FA conversion.

**Table 2:** Kinectic Parameter for 50% FA Conversion Using [RuCl_2_(PNP)] as a Catalyst^a^^b^^c^

Entry	Temp (°C)	Time (h)	TON	TOF (h^–1^)	*k*_obs_ × 10^–2^ (min^.–1^)
**1**	60	0.81	590	728	6.85
**2**	70	0.77	590	766	11.05
**3**	75	0.35	590	1685	22.34
**4**	80	0.21	590	2809	39.47
**5**	90	0.10	590	5900	50.89
**6**	100	0.09	590	6555	70.83

a Solvent = HMDSO (10 mL) containing THF (1 mL).

b Molar ratio Ru/FA/NEt3 = 1/1204/843.

c [Ru] loaded = 0.08% related to FA.

**Figure 4:**
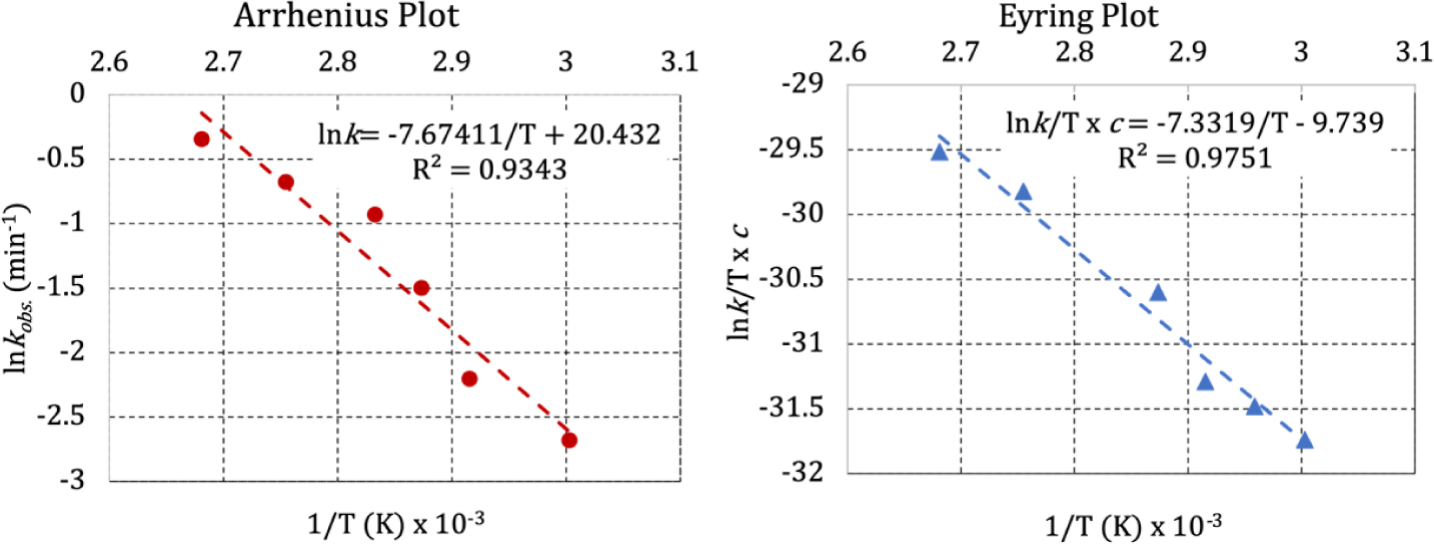
Arrhenius and Eyring plot at the temperature range of 60–100 °C, due to the catalyst performance of **1** in the decomposition of FA.

It is interesting to note that at 100 °C, the catalytic performance of **1** was 10-fold faster than the performance at 60 °C. The *k*_obs_ values on the temperature range described in Table 2 were used in the Arrhenius and Eyring plots (Figure 4), providing the activation energy as *E*_a_ = 64.0 kJ mol^–1^.

This result fits well with results previously reported; Dupont and coworkers^5^ observed *E*_a_ = 69.1 kJ mol^–1^ using [{RuCl_2_(*p*-cymene)}_2_] dissolved in the ionic liquid for decomposing FA, while Singh and coworkers^25^ reported *E*_a_ = 87.9 k*J* mol^–1^, using a half-sandwich cationic complex [(η^6^-C_6_H_6_)Ru(κ^2^-NpyNHMe-8-AmQ)Cl]^+^ as a catalyst.

From the Eyring plot, the activation entropy was obtained as Δ*S*‡ = −9.7 e.u., showing a transition state slightly lower entropic than the initial state. Care must be taken when interpreting the mechanism for reactions that have Δ*S*‡ between −10 and +10 e.u. because solvent reorganization can also contribute, especially for polar solvents and charged metal complexes.^44^ However, HMDSO is a very nonpolar solvent, and the target complex **1** is a neutral metal complex. It suggests, in the case of FA decomposition catalyzed by **1**, a mechanism toward a concerted pathway. The activation variation enthalpy corroborates this idea, Δ*H*‡ = 61.0 k*J* mol^–1^, which is low in the case of some coordination ligands in the core sphere of **1**. The activation Gibbs variation energy was obtained as Δ*G*‡ = 13.35 kJ mol^–1^, suggesting nonspontaneous reactions.

**Figure 5:**
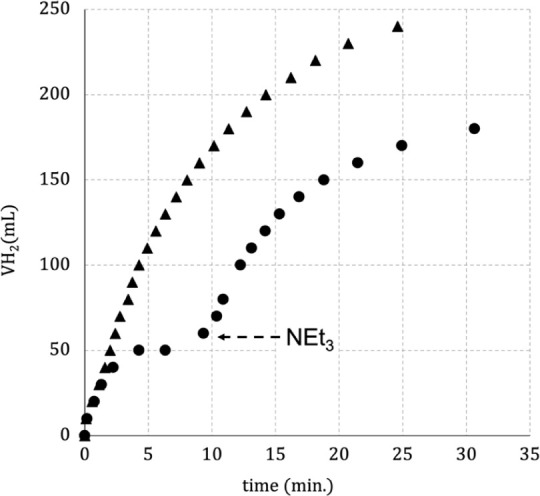
Performance of [RuH_2_(PNP)] (**2**) on the FA decomposition, started with NEt_3_ (triangle) and started without NEt_3_ (circle).

In an attempt to promote FA decomposition without NEt_3_, the *in situ* complex [RuH_2_(PNP)] (**2**) generated from **1** was applied in an HMDSO solution with KBEt_3_H (Figure 5).

The catalytic performance of **2** on the FA decomposition showed total conversion within 0.41 h at 90 °C and TOF = 2938 h^–1^, while complex **1**, under the same conditions, achieved total conversion in 0.26 h and TOF = 4631 h^–1^. In other words, complex **1** is almost twice as fast as **2**. However, as mentioned above, complex **1** does not act as a catalyst for FA decomposition without NEt_3_, and it must be added at the beginning of the reaction.

In contrast, complex **2** started the reaction without NEt_3_ (Figure 5, circle), producing H_2_ up to a maximum: 60 mL or 24% of H_2_; TON = 289; TOF = 1926 h^–1^. Then, NEt_3_ (7.0 mmol) was added to the system, and the reaction restarted, producing a further amount of H_2_: 210 mL or 85% of H_2_, TON = 1032; TOF = 782 h^–1^. These results suggest that NEt_3_ participates herein as a cocatalyst on FA decomposition and plays a significant role in the process of H_2_ production from FA.

## Conclusion

The complex [RuCl_2_(PNP)] (**1**) has been synthesized from the well-known binuclear precursor [RuCl_2_(*p*-cym)]_2_ and characterized as a five-coordinate coordination complex. Complex **1** catalyzed the decomposition of formic acid (FA) and ammonium formate (AF), separately or in a mixture of both. FA and AF decomposition produced H_2_ + CO_2_ and H_2_ + CO_2_ + NH_3_, respectively. H_2_ was identified by volumetric analysis in the presence of a hydrogen acceptor (cyclohexene). A concomitantly catalyzed THF polymerization reaction was observed in the decomposition of AF. A white solid, identified as carbamic acid, was also observed in the AF decomposition or in the decomposition of the FA/AF mixture due to the reaction between NH_3_ + CO_2_. In the case of FA decomposition catalyzed by **1**, a kinetic study suggests a more associative mechanism than the mechanism catalyzed by other complexes under similar conditions. THF was found to be the best solvent for AF decomposition, while HMDSO was the best solvent for **1**-catalyzed FA decomposition. The complex [Ru(H)_2_(PNP)] (**2**), prepared *in situ*, catalyzed the decomposition of FA but gave rise to a catalytic activity two times lower than that of **1**. In contrast, **2** initiated FA decomposition without a base, while **1** needed the Brønsted–Lowry base from the beginning. NEt_3_ plays a significant role in the process of producing H_2_ from the decomposition of FA, and therefore must participate herein as a cocatalyst of the reaction.

## Supporting Information Available

A physicochemical characterization of [RuCl_2_(PNP), including NMR (^1^H, ^13^C, ^1^H–^13^C HSQC, ^1^H–^13^C HMBC, and ^31^P{^1^H}), FTIR-ATR, mass spectroscopy (LIFDI), cyclic voltammetry, and UV/vis data; a detailed description of the reactivity of the [RuCl_2_(PNP)] complex with KBEt_3_H and NH_4_HCO_2_, which was accompanied by ^1^H and ^31^P{^1^H} NMR experiments, suggesting an *in situ* preparation of [RuCl(H)(PNP)], [RuH_2_(PNP)], and [RuCl(OCOH)(PNP)] complexes; lastly, the DFT results of the target complex, as well as the catalysis gadgets, and characterization of carbamic acid are presented in detail (PDF)
