# Supplementary material for: Hydrogen Production by the Ruthenium(II) Complex Bearing a Bulky PNP Ligand: A Catalyst for the Decomposition of Formic Acid and/or Ammonium Formate
Source: ACS Omega. 2024 Dec 11;9(51):50758–65. doi: 10.1021/acsomega.4c09025 (PMC11683645; doi:10.1021/acsomega.4c09025)
Supplement: Supplementary file 1 — ao4c09025_si_001.pdf [file ao4c09025_si_001.pdf]

Supporting Information Material

Hydrogen production by ruthenium (II) complex bearing a bulky PNP-ligand: a catalyst for the decomposition of formic acid and/or ammonium formate

André L. Bogado*[a], Leon Kambiz Paschai Darian[b], David Bürgy[b], Lucas da Silva dos Santos[c], *Leonardo T. Ueno*[d].

[a]*Instituto de Ciências Exatas e Naturais do Pontal, Universidade Federal de Uberlândia, CEP 38304-402, Ituiutaba - MG, Brazil*

[b]*Anorganisch-Chemisches Institut, Universität Heidelberg, Im Neuenheimer Feld 270, 69120 Heidelberg, Germany*

[c]*Instituto de Química, Universidade Federal de Uberlândia, CEP 38400-902, Uberlândia - MG, Brazil*

[d]*Departamento de Química, Instituto Tecnológico de Aeronáutica, CEP 12228-900, São José dos Campos - SP, Brazil*

Table of Contents

Characterization of [$RuCl_2$(PNP)] 3

*Fig. S 1: ^1H NMR of [$RuCl_2$(PNP)] (600.16 MHz, C_6D_6, rt; # = ^{13}C satellite from solvent, * = unknown impurity, $ = grease).* 3

*Fig. S 2: ^{13}C NMR of [$RuCl_2$(PNP)] (150.90 MHz, C_6D_6, rt, * = unknown impurity, $ = grease).* 4

Fig. S 3: ^1H-^{13}C HSQC NMR of [$RuCl_2$(PNP)] (600.16/150.90 MHz, C_6D_6, 295 K). 4

Fig. S 4: ^1H-^{13}C HMBC NMR of [$RuCl_2$(PNP)] (600.16/150.90 MHz, C_6D_6, 295 K). 5

Fig. S 5: $^{31}P\{^1H\}$ NMR of [$RuCl_2$(PNP)] (242.9 MHz, C_6D_6, rt). 5

Fig. S 6: FTIR-ATR of precursor, free ligand and [$RuCl_2$(PNP)] from 400 – 4000 cm^{-1}. 6

Fig. S 7: Mass spectroscopy (LIFDI): m/z (%). 6

Fig. S 8 . A) CV of the [$RuCl_2$(PNP)] in THF solution: [Ru] = 5 mmol L^{-1}; [n-Bu_4NPF_6] = 0.1 mol L^{-1}; scanned anodically from -1.0 to 0.0 V. Glassy carbon disc (WE), Pt wire (CE), Ag wire (RE). B) I vs square-root of the potential sweep rate. 7

Fig. S 9: A) Uv/vis of the [$RuCl_2$(PNP)] complex in THF solution: a = 0.5×10^{-3}, b = 2.5×10^{-4}, c = 1.3×10^{-4}, d = 6.3×10^{-5} and e = 3.1×10^{-5} mol L^{-1}. B) Molar absorptivity determination. .7

Reactivity of [$RuCl_2$(PNP)] 8

In situ preparation of [RuCl(H)(PNP)] and [RuH_2(PNP)] 8

Fig. S 10: $^{31}P\{^1H\}$ NMR and ^1H NMR data, due to the reactivity of the [$RuCl_2$(PNP)] with $KBEt_3H$. 8

In situ preparation of [RuCl(OCOH)(PNP)] 9

Fig. S 11: $^{31}P\{^1H\}$ NMR and ^1H NMR data, due to the reactivity of the [$RuCl_2$(PNP)] with NH_4HCO_2 (AF) 9

DFT Calculation 10

*Table S 1: Atomic numbering, bond length and angles of the optimized structure of **1**, using five different functional bases in DFT calculation.* 10

*Fig. S 12: HOMO and LUMO orbitals of the optimized structure of **1**, using A) B3LYP, B) CAM-B3LYP, C) LC-wPBE and D) PBE0 functional in THF. LANL2DZ basis for ruthenium, 6-31G for carbon and hydrogen, and 6-31G(d,p) for the remaining atoms.* 11

Catalysis 12

Fig. S 13: Gadget used in the decomposition reaction of AF and FA to measure the amount of H_2. 12

Fig. S 14: Gadget used in the hydrogenation of cyclohexene. 12

Characterization of Carbamic Acid 13

Scheme S. 1: Carbamic acid obtained from the reaction of NH_3 and CO_2. 13

Fig. S 16: TGA of the white specimen (carbamic acid) from 30 - 60 °C, 10K/min. in N_2 atmosphere (50 mL / min.). 14

Fig. S 17: FTIR-ATR from the white specimen (carbamic acid) compared with ammonium carbonate from 400 – 4000 cm^{-1}. 14

Fig. S 18: ^1H NMR data of the white specimen (carbamic acid) (600.1 MHz, MeOH-d_4, rt). ..15

Fig. S 19: ^{13}C NMR data of the white specimen (carbamic acid) (150.9 MHz, MeOH-d_4, rt). .15

Characterization of [$RuCl_2$(PNP)]

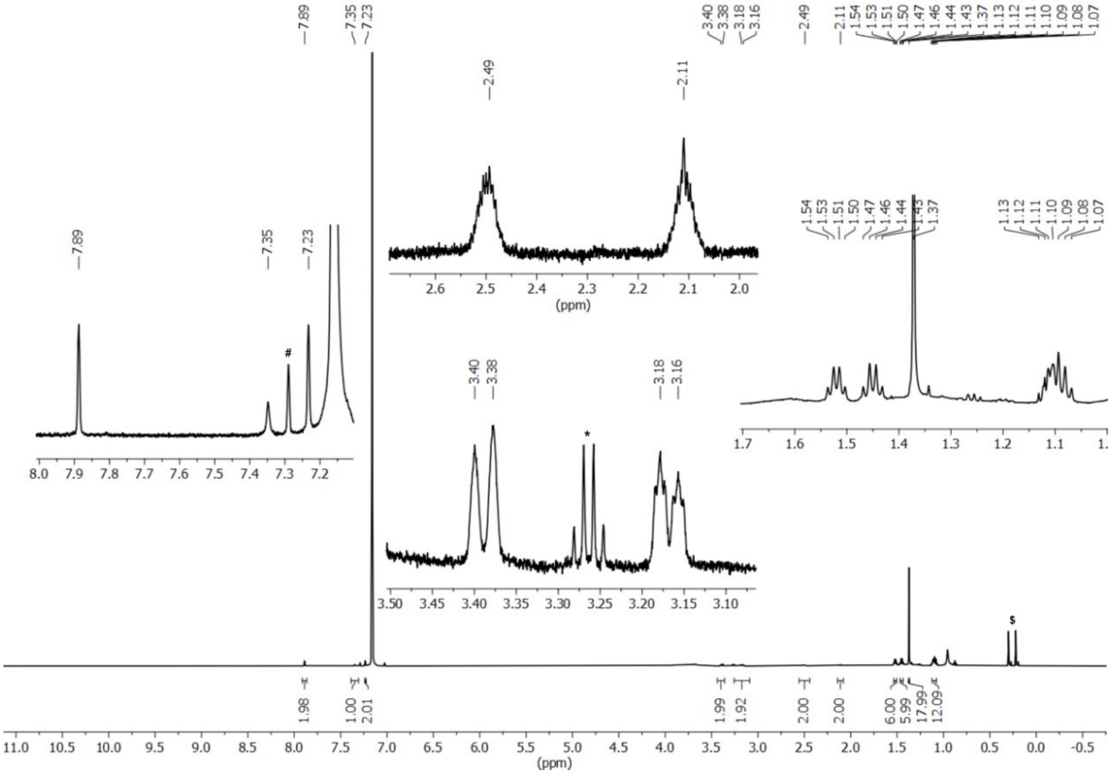

Fig. S 1: ^{1}H NMR of [$RuCl_2$(PNP)] (600.16 MHz, C_6D_6, rt; # = ^{13}C satellite from solvent, * = unknown impurity, $ = grease).

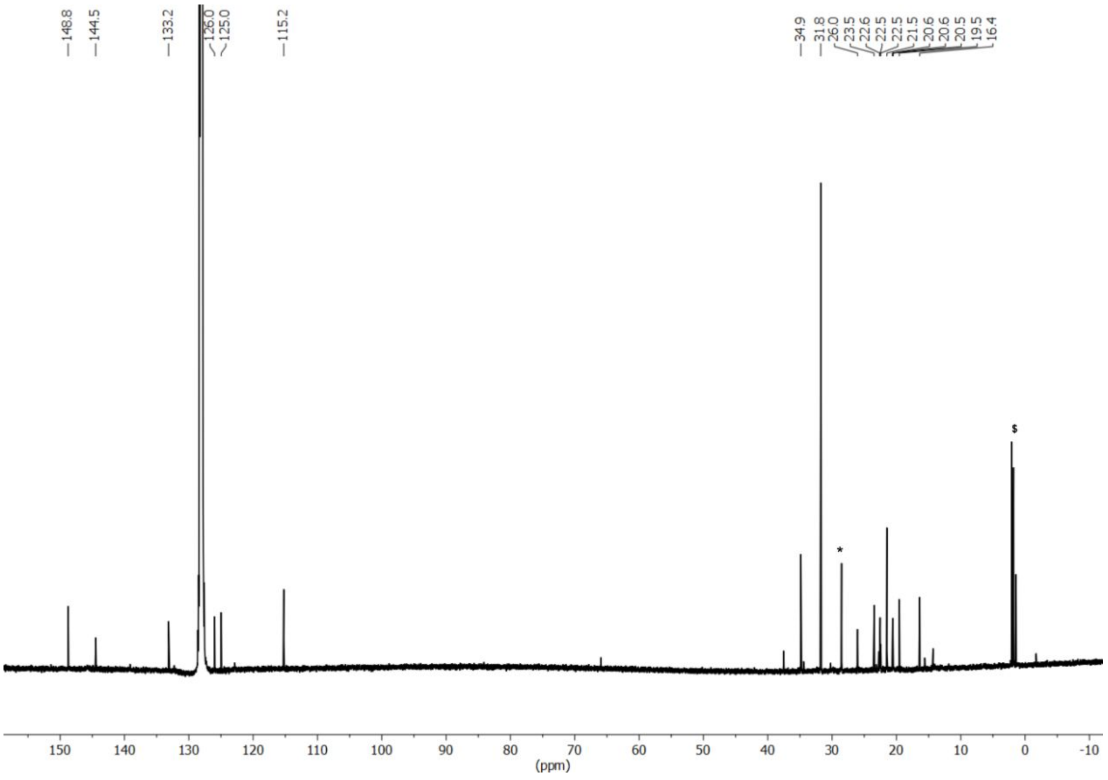

Fig. S 2:^{13}C NMR of [RuCl$_2$(PNP)] (150.90 MHz, C$_6$D$_6$, rt, * = unknown impurity, $ = grease).

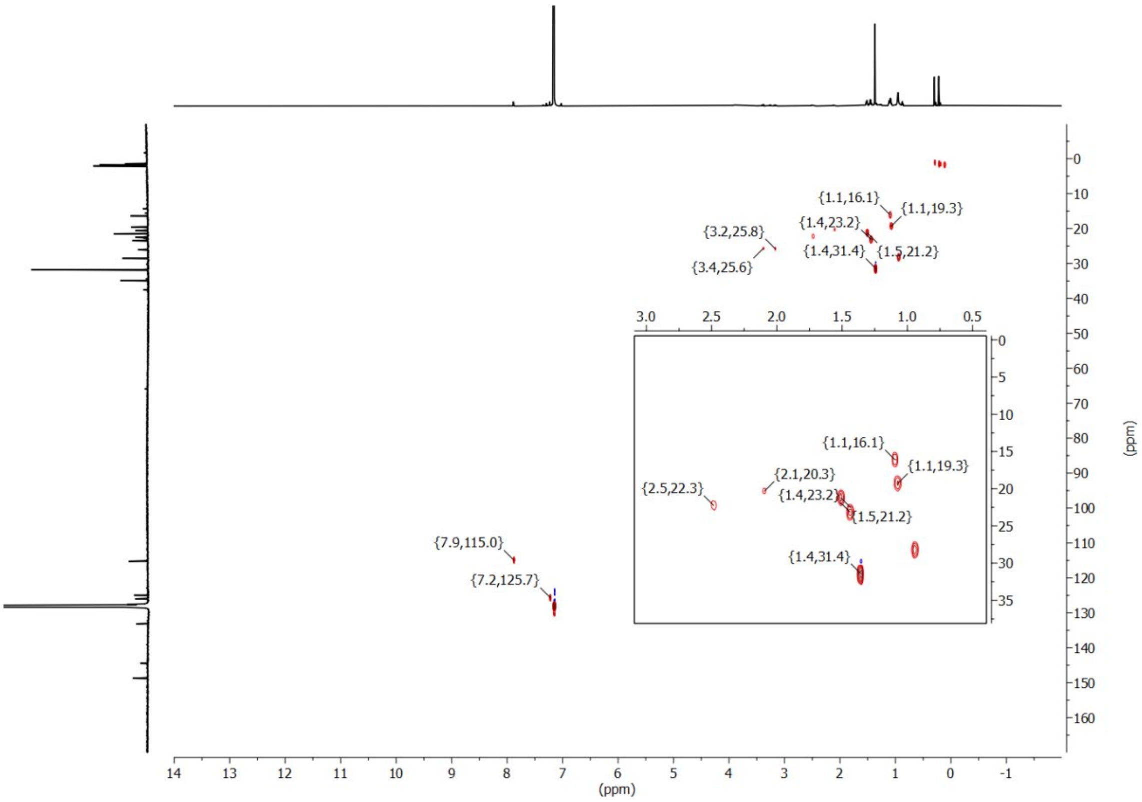

Fig. S 3: ^{1}H-^{13}C HSQC NMR of [RuCl$_2$(PNP)] (600.16/150.90 MHz, C$_6$D$_6$, 295 K).

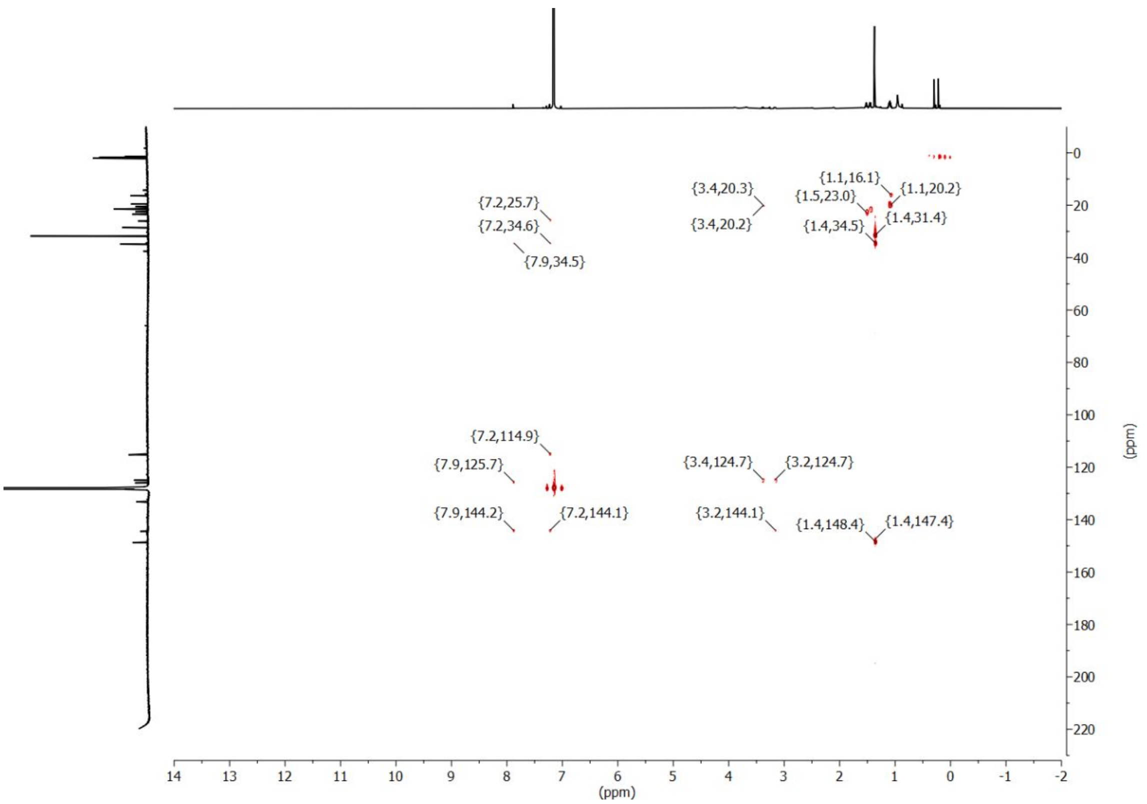

Fig. S 4: ^{1}H-^{13}C HMBC NMR of [$RuCl_2$(PNP)] (600.16/150.90 MHz, C_6D_6, 295 K).

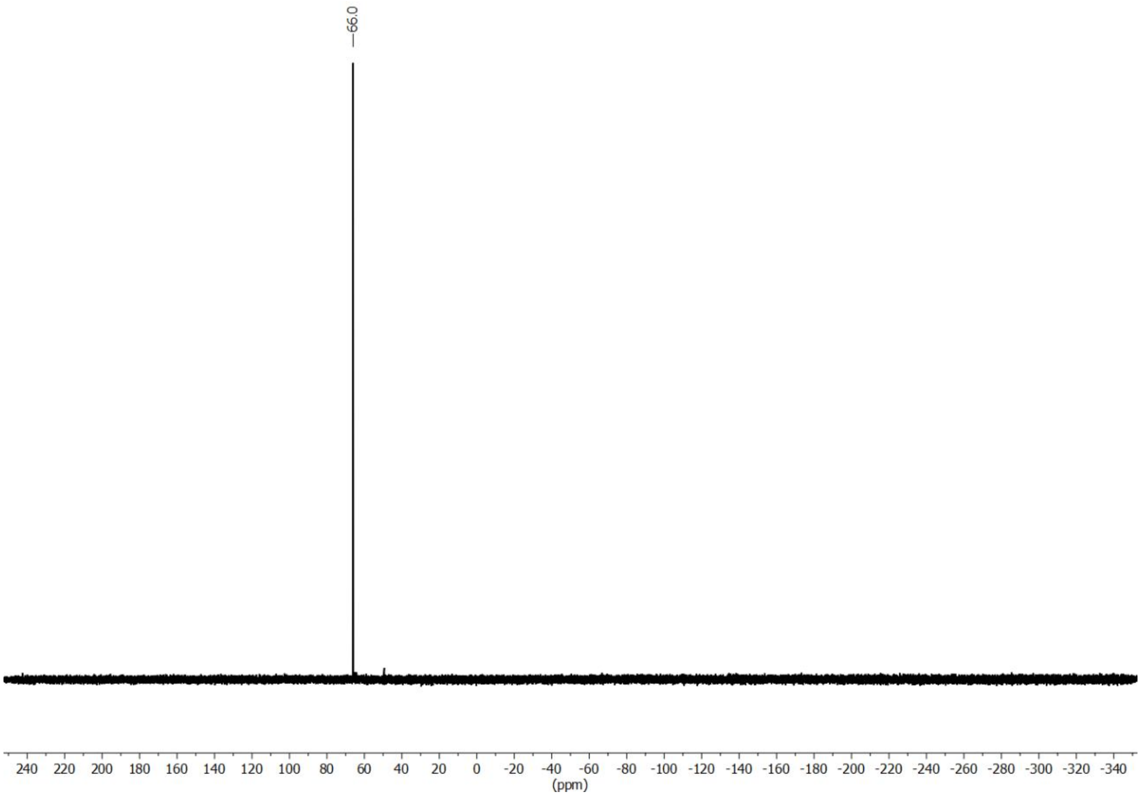

Fig. S 5: ^{31}P{^{1}H} NMR of [$RuCl_2$(PNP)] (242.9 MHz, C_6D_6, rt).

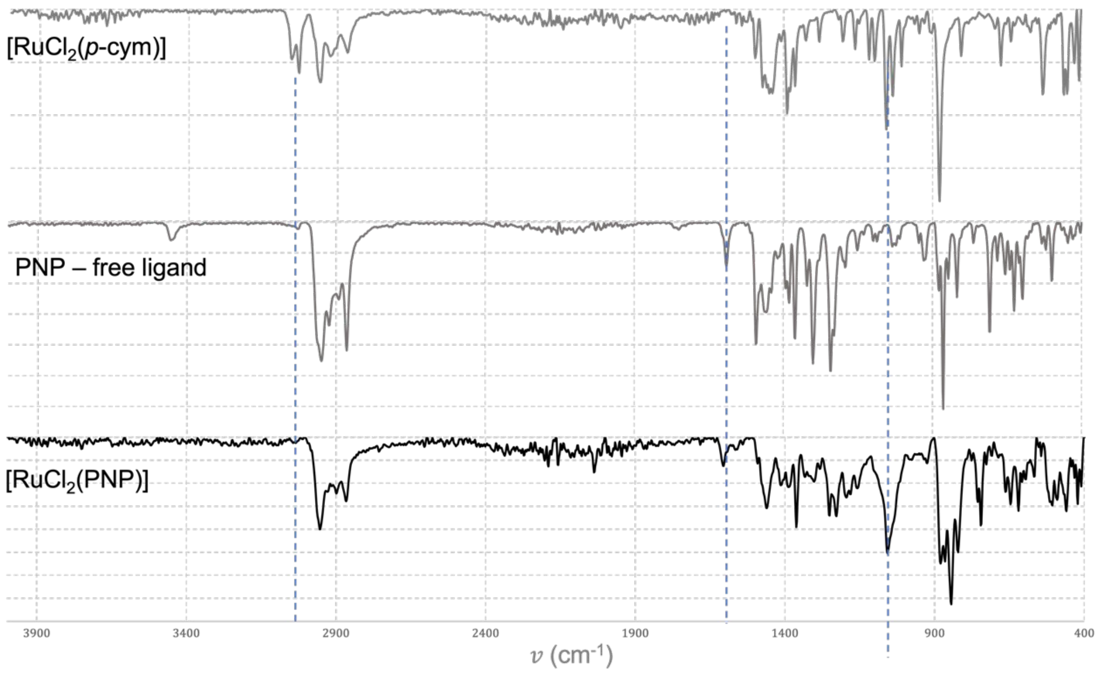

Fig. S 6: FTIR-ATR of precursor, free ligand and [$RuCl_2$(PNP)] from 400 – 4000 cm^{-1}.

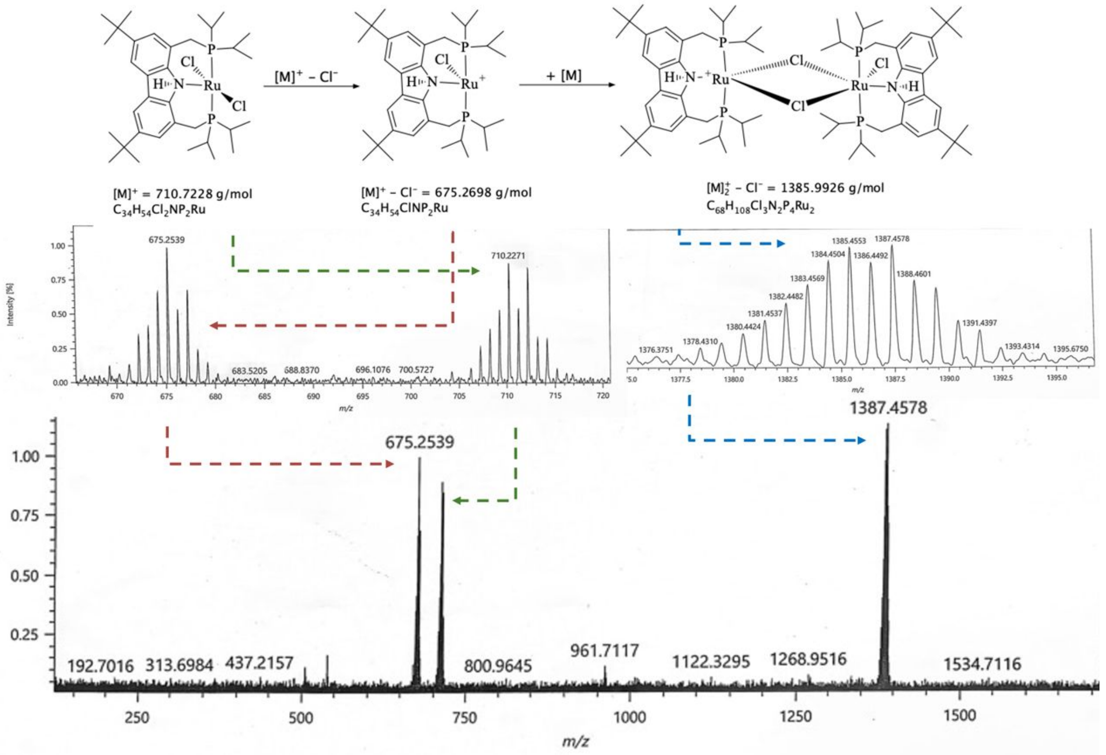

Fig. S 7: Mass spectroscopy (LIFDI): m/z (%).

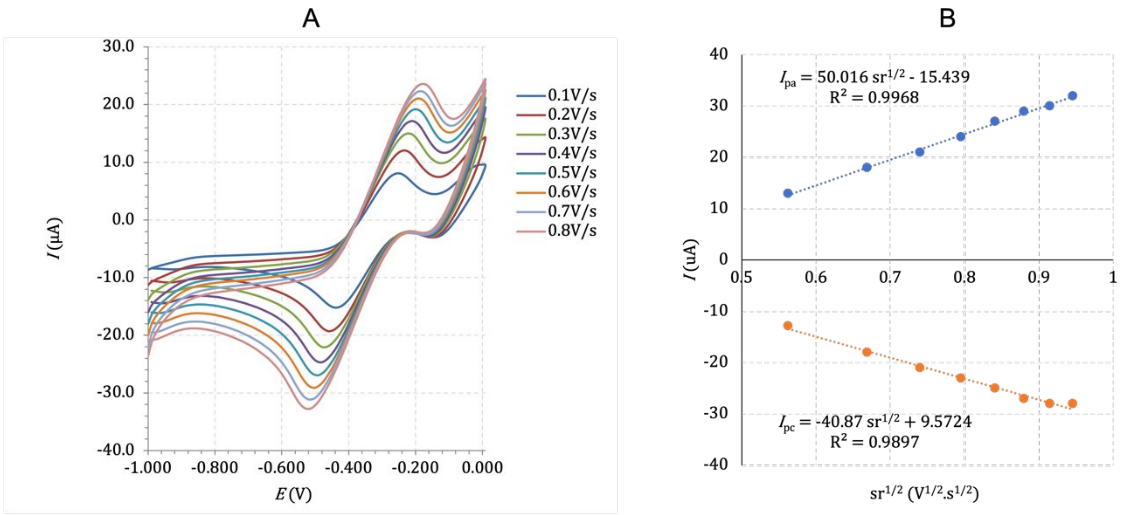

Fig. S 8 . A) CV of the [$RuCl_2$(PNP)] in THF solution: [Ru] = 5 mmol L^{-1}; [n-Bu_4NPF_6] = 0.1 mol L^{-1}; scanned anodically from -1.0 to 0.0 V. Glassy carbon disc (WE), Pt wire (CE), Ag wire (RE). B) I vs square-root of the potential sweep rate.

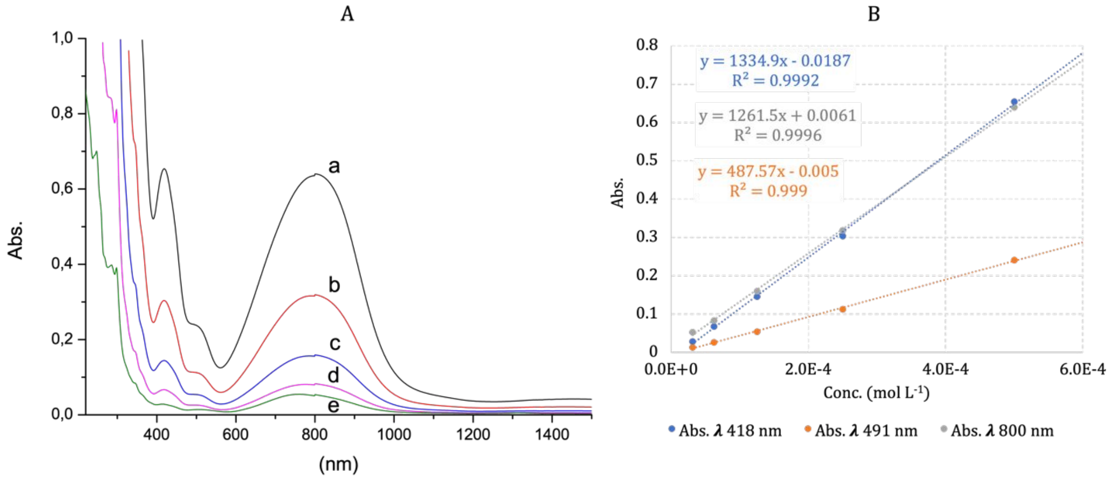

Fig. S 9: A) Uv/vis of the [$RuCl_2$(PNP)] complex in THF solution: a = 0.5 × 10^{-3}, b = 2.5 × 10^{-4}, c = 1.3 × 10^{-4}, d = 6.3 × 10^{-5} and e = 3.1 × 10^{-5} mol L^{-1}. B) Molar absorptivity determination.

Reactivity of [$RuCl_2$(PNP)]

In situ preparation of [RuCl(H)(PNP)] and [RuH_2(PNP)]

Inside an NMR tube, the complex **1** (10 mg; 14.0 μmol) was dissolved in THF-d_8 (0.6 mL), and $KBEt_3H$ (21.5 μL, 140 μmol) was added. The color immediately changed from green to yellowish orange. The mixture was allowed to react for 1h at room temperature before subsequent NMR spectroscopy analysis. ^1H NMR (600.1 MHz, THF-d_8, rt) δ (ppm): - 9.25 (*t*, J_{HP} = 16.4 Hz, (PNP)Ru-H). $^{31}P\{^1H\}$ NMR (242.9 MHz, THF-d_8, rt) δ (ppm): 45 (s, [RuClH(PNP)]. Then, the reaction was heated at 70°C for 16 h, and the color changed from yellowish orange to yellow. ^1H NMR (600.1 MHz, THF-d_8, rt) δ (ppm): -9.2 (m, [RuH_2(PNP)]). $^{31}P\{^1H\}$ NMR (242.9 MHz, THF-d_8, rt) δ (ppm): 85.0 (s, [RuH_2(PNP)]).

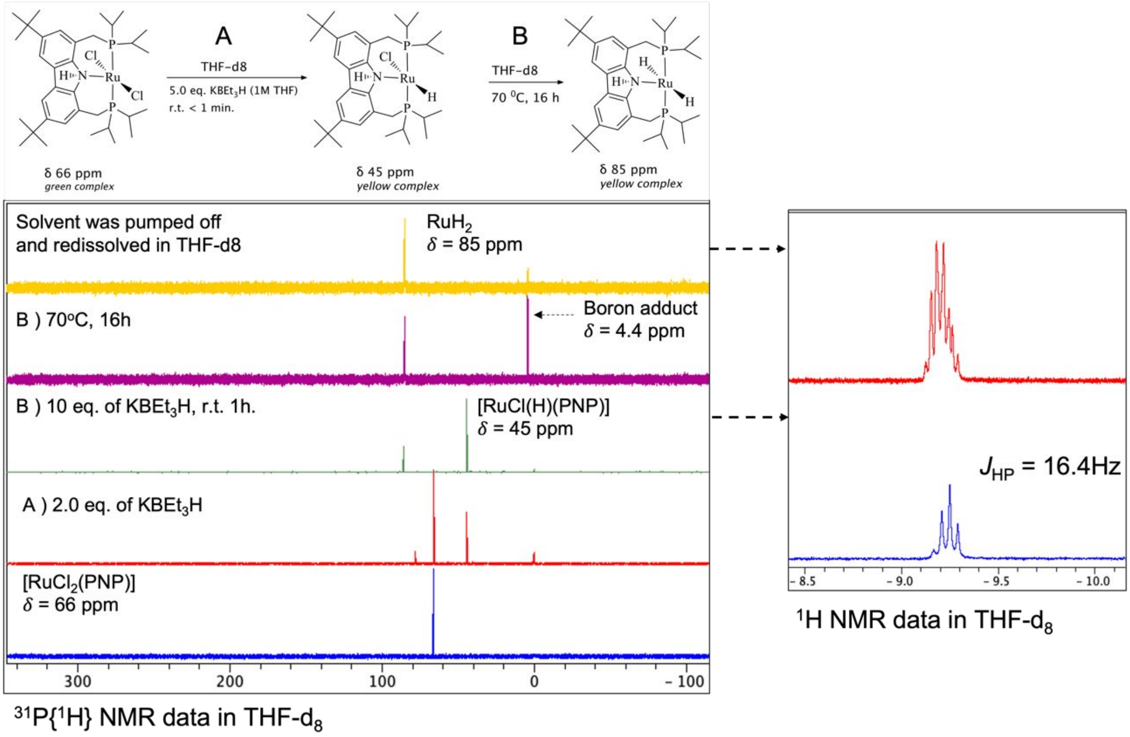

Fig. S 10: $^{31}P\{^1H\}$ NMR and ^1H NMR data, due to the reactivity of the [$RuCl_2$(PNP)], A) with 2.0 eq of $KBEt_3H$, and B) 10 eq. of $KBEt_3H$, time and temperature variation.

In situ preparation of [RuCl(OCOH)(PNP)]

Inside an NMR tube, the complex **1** (10 mg; 14.0 μmol) was dissolved in THF-d_8 (0.6 mL), and NH_4OCOH (8.8 mg, 140 μmol) was added. The reaction was heated at 70°C for 16 h, and the color changed from green to greenish brown, with the release of gas. ^1H NMR (600.1 MHz, THF-d_8, rt) δ (ppm): 10.93 (s, [RuCl(OCO<u>H</u>)(PNP)]. $^{31}P\{^1H\}$ NMR (242.9 MHz, THF-d_8, rt) δ (ppm): 69.8.

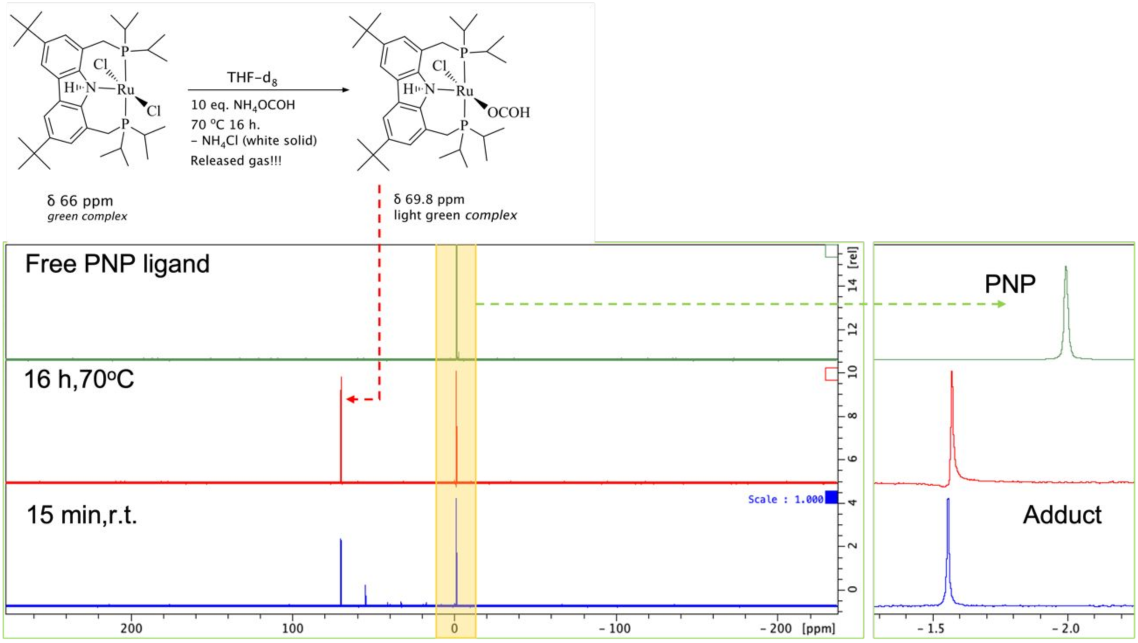

Fig. S 11: $^{31}P\{^1H\}$ NMR and ^1H NMR data, due to the reactivity of the $[RuCl_2(PNP)]$ with NH_4HCO_2 (AF)

DFT Calculation

Table S 1: Atomic numbering, bond length and angles of the optimized structure of **1**, using five different functional bases in DFT calculation.

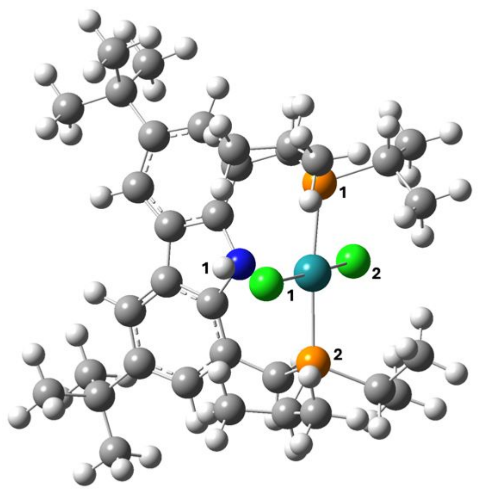

	Functional Base				
Bond length (Å)	B3LYP	B3PW91	CAM-B3LYP	LC-wPBE	PBE0
Ru-Cl1	2.510	2.473	2.485	2.449	2.464
Ru-Cl2	2.492	2.457	2.469	2.439	2.447
Ru-P1	2.418	2.383	2.404	2.381	2.376
Ru-P2	2.416	2.387	2.408	2.378	2.377
Ru-N	2.147	2.119	2.134	2.116	2.112
N-H1	1.034	1.035	1.031	1.032	1.035
Angles (°)					
Cl1-Ru-Cl2	178.9	178.4	178.7	178.1	178.0
Cl1-Ru-P1	91.2	90.9	91.1	91.1	91.0
Cl1-Ru-P2	91.2	91.2	91.2	91.6	91.4
Cl2-Ru-P1	88.7	88.9	88.8	88.8	88.8
Cl2-Ru-P2	88.8	88.9	88.8	88.4	88.7
Cl1—Ru-N	86.9	87.6	87.2	87.9	87.3
Cl2-Ru-N	91.9	90.7	91.5	90.2	90.7
P1-Ru-P2	174.9	174.7	175.3	174.9	174.6
P1-Ru-N	92.2	92.0	91.8	91.8	92.0
P2-Ru-N	92.2	92.7	92.2	92.5	92.7
Ru-N-H1	86.6	86.8	87.3	88.0	87.0

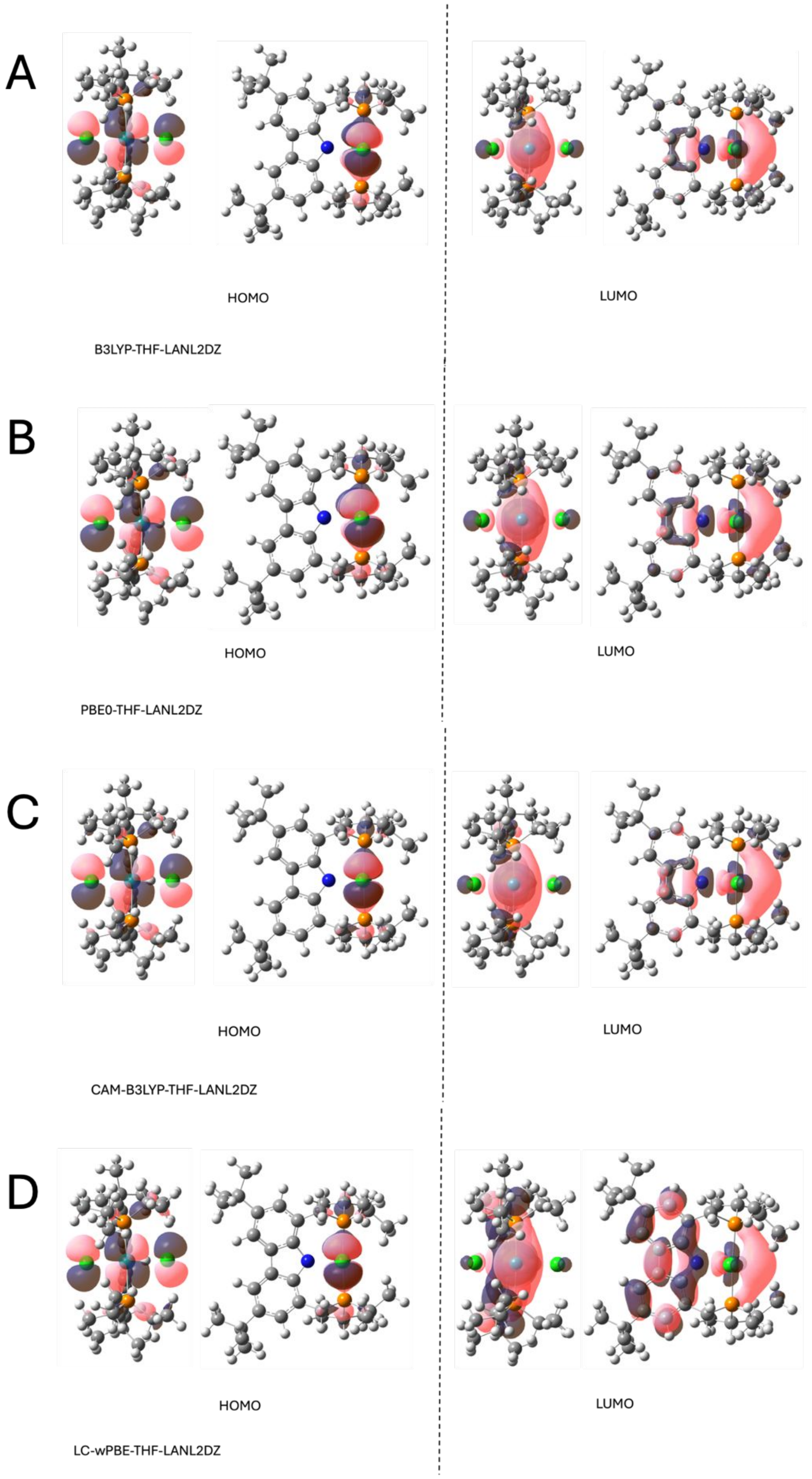

Fig. S 12: HOMO and LUMO *orbitals* of the optimized structure of **1**, using A) B3LYP, B) CAM-B3LYP, C) LC-wPBE and D) PBE0 functional in THF. LANL2DZ basis for ruthenium, 6-31G for carbon and hydrogen, and 6-31G(d,p) for the remaining atoms.

Catalysis

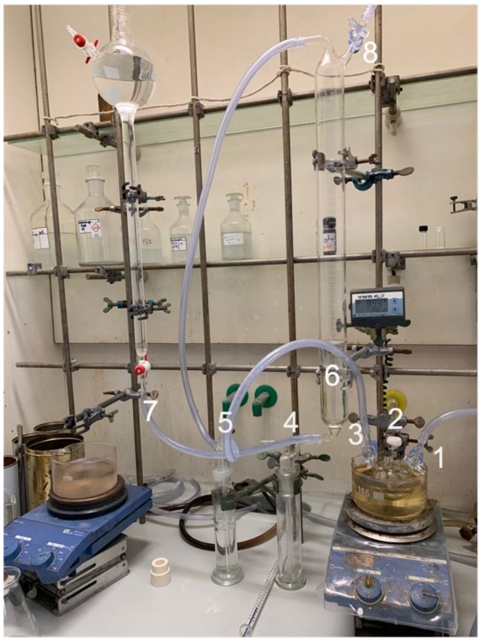

1. Argon inlet
2. Input / output of samples
3. Gas outlet
4. Trap of H_2SO_4 (Only for AF decomposition)
5. Trap of NaOH (8 mol L^{-1})
6. Graduated water column (500 mL)
7. Water tank
8. Depressurization of system

Fig. S 13: Gadget used in the decomposition reaction of AF and FA to measure the amount of H_2.

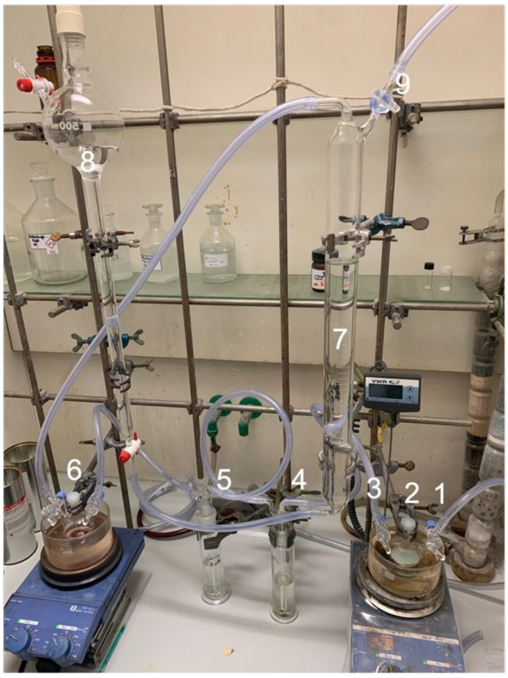

1. Argon inlet
2. Input / output of samples
3. Gas outlet
4. Trap of H_2SO_4 (Only for AF decomposition)
5. Trap of NaOH (8 mol L^{-1})
6. Hydrogenation of C-ene (2nd reaction)
7. Graduated water column (500 mL)
8. Water tank
9. Depressurization of system

Fig. S 14: Gadget used in the hydrogenation of cyclohexene.

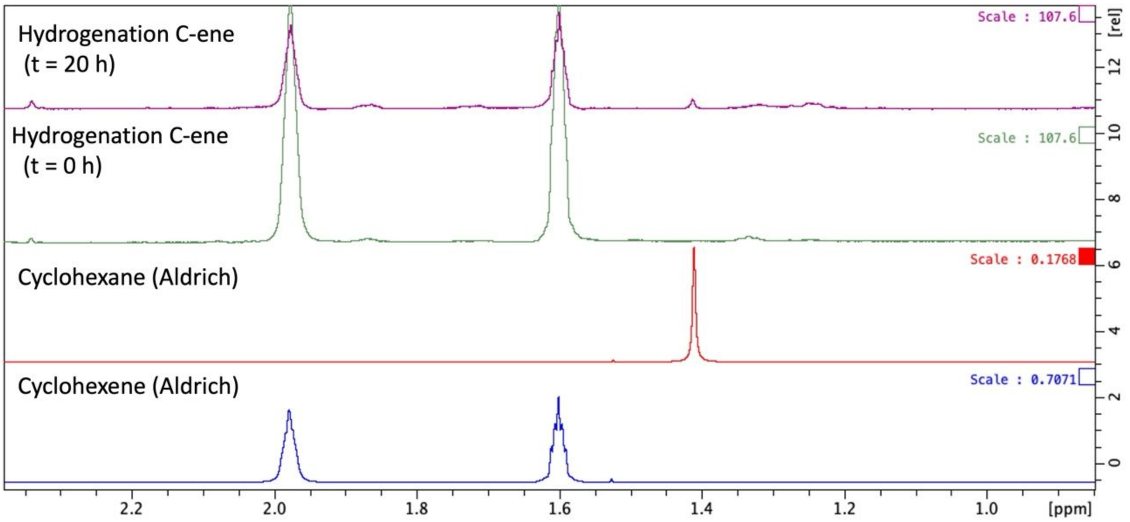

Fig. S 15: ^{1}H NMR data related to the use of produced H_2 in the hydrogenation of cyclohexene (600.1 MHz, $CDCl_3$, rt)

Characterization of Carbamic Acid

The white specimen obtained during the decomposition reaction of the ammonium formate catalyzed by [$RuCl_2$(PNP)] in HDMSO solution was characterized by TGA, FTIR-ATR, ^{1}H and ^{13}C NMR data. TGA of the white solid reveals only one process of decomposition from 100 to 0 % of mass, suggesting an organic compound. FTIR-ATR has shown a broad band in the right of the range of 3300 – 2600 cm^{-1}, which is typical for amine salts, while ammonium salts absorb in the left of this range. The carboxylate band is also present in the FTIR data, centered in 1400 cm^{-1}, as well as a thin stretch for N-H bound at 772 cm^{-1}. The ^{1}HNMR data in MeOH-d_4 has shown two singlet signals at 5.3 and 8.5 ppm, which is consistent with NH_2 and OH groups. The ^{13}C NMR data in DMSO-d_6 provided only one singlet signal at 166 ppm, which is plausible for a carbonyl group. This physicochemical characterization suggests the presence of carbamic acid (Scheme 1 S).

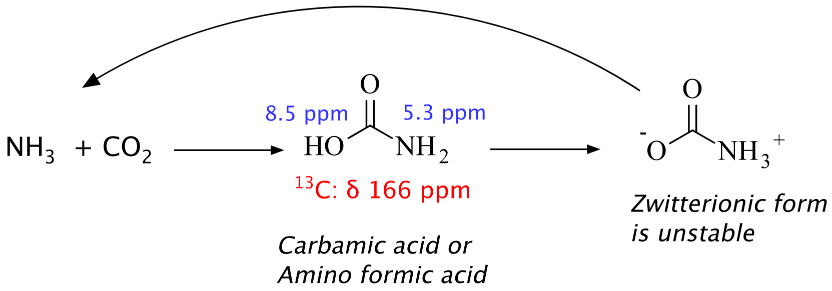

Scheme S. 1: Carbamic acid obtained from the reaction of NH_3 and CO_2.

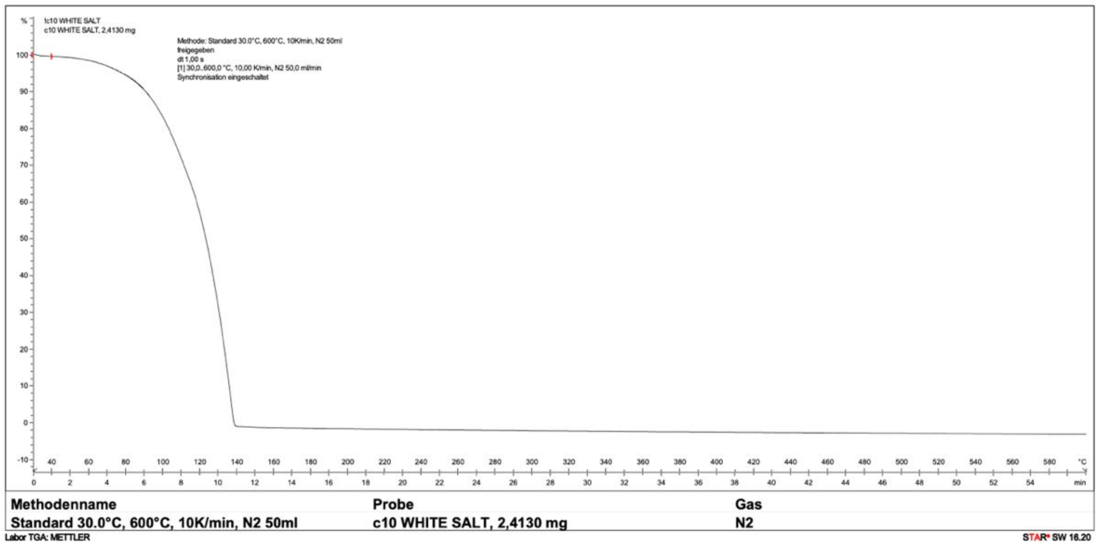

Fig. S 16: TGA of the white specimen (carbamic acid) from 30 - 60°C, 10K/min. in N_2 atmosphere (50 mL / min.).

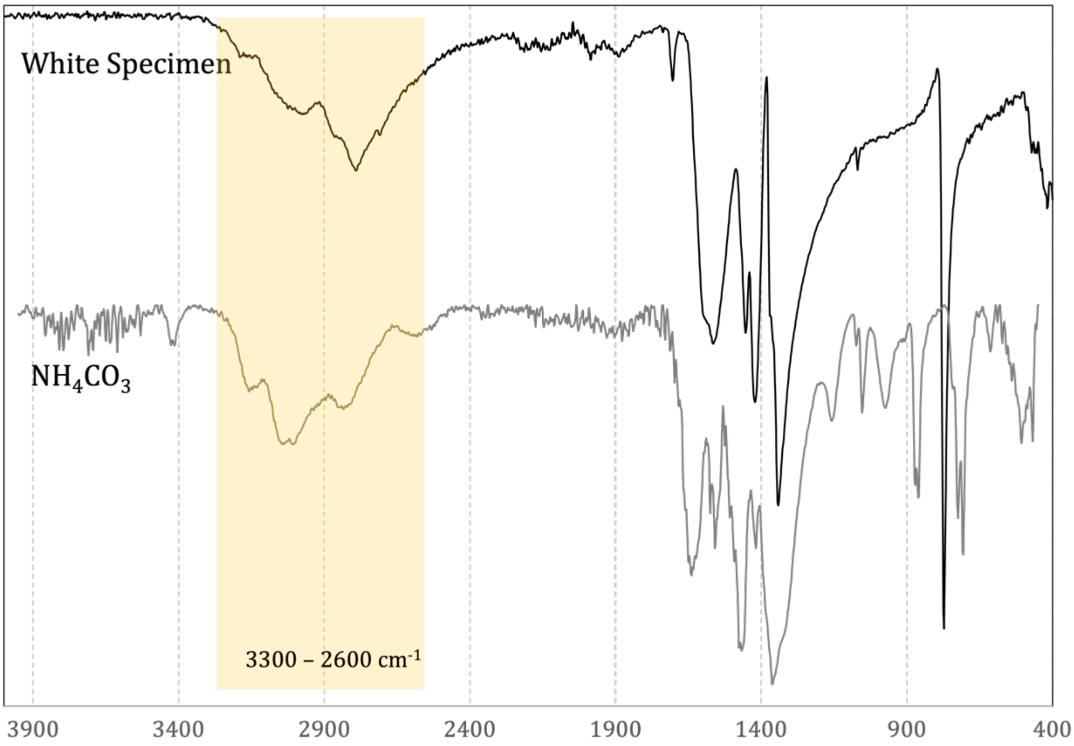

Fig. S 17: FTIR-ATR from the white specimen (carbamic acid) compared with ammonium carbonate from 400 – 4000 cm^{-1}.

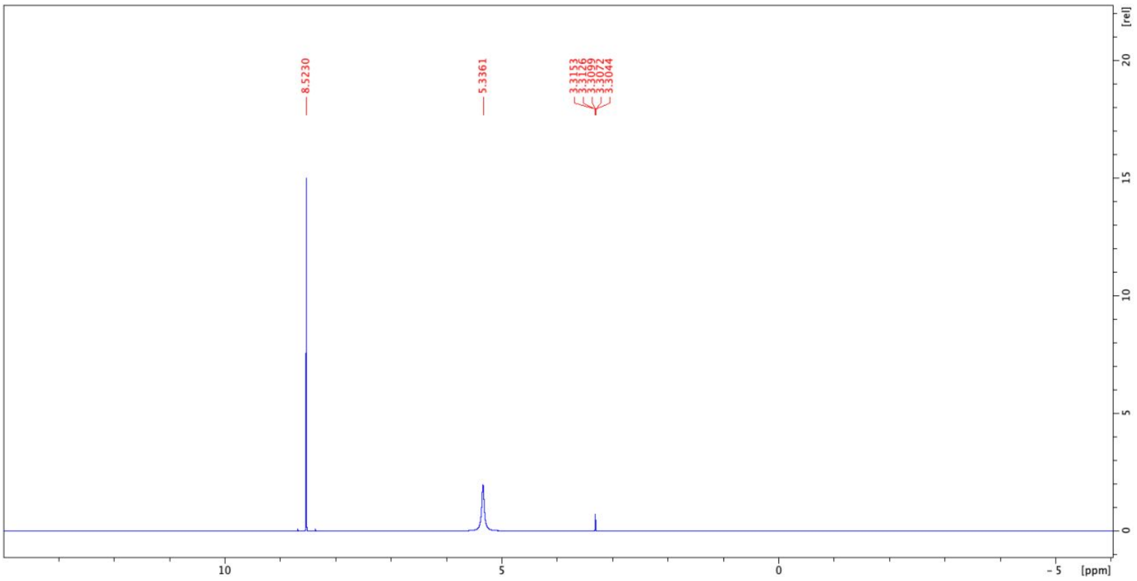

Fig. S 18: ^{1}H NMR data of the white specimen (carbamic acid) (600.1 MHz, MeOH-d_4, rt).

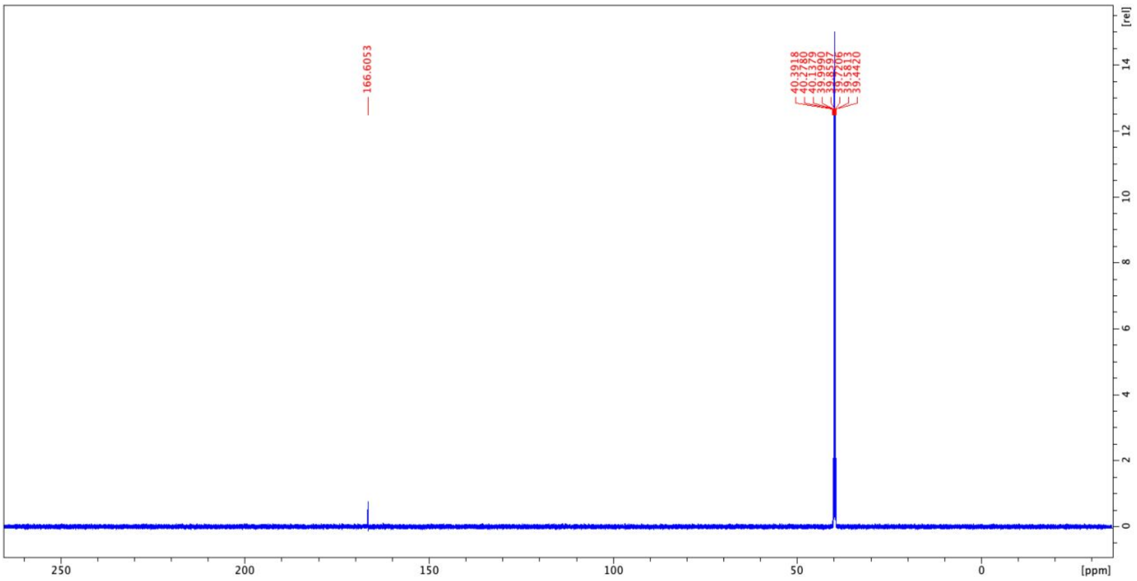

Fig. S 19: ^{13}C NMR data of the white specimen (carbamic acid) (150.9 MHz, MeOH-d_4, rt).
